# Inferential Structure Determination of Chromosomes from Single-Cell Hi-C Data

**DOI:** 10.1371/journal.pcbi.1005292

**Published:** 2016-12-27

**Authors:** Simeon Carstens, Michael Nilges, Michael Habeck

**Affiliations:** 1 Unité de Bioinformatique Structurale, Department of Structural Biology and Chemistry, Institut Pasteur, Paris, France; 2 Statistical Inverse Problems in Biophysics, Max Planck Institute for Biophysical Chemistry, Göttingen, Germany; 3 Felix Bernstein Institute for Mathematical Statistics in the Biosciences, University of Göttingen, Göttingen, Germany; Rutgers University, UNITED STATES

## Abstract

Chromosome conformation capture (3C) techniques have revealed many fascinating insights into the spatial organization of genomes. 3C methods typically provide information about chromosomal contacts in a large population of cells, which makes it difficult to draw conclusions about the three-dimensional organization of genomes in individual cells. Recently it became possible to study single cells with Hi-C, a genome-wide 3C variant, demonstrating a high cell-to-cell variability of genome organization. In principle, restraint-based modeling should allow us to infer the 3D structure of chromosomes from single-cell contact data, but suffers from the sparsity and low resolution of chromosomal contacts. To address these challenges, we adapt the Bayesian Inferential Structure Determination (ISD) framework, originally developed for NMR structure determination of proteins, to infer statistical ensembles of chromosome structures from single-cell data. Using ISD, we are able to compute structural error bars and estimate model parameters, thereby eliminating potential bias imposed by *ad hoc* parameter choices. We apply and compare different models for representing the chromatin fiber and for incorporating singe-cell contact information. Finally, we extend our approach to the analysis of diploid chromosome data.

## Author Summary

Spatial interactions between distant genomic regions are of fundamental importance in gene regulation and other nuclear processes. Recent chromatin crosslinking (“Hi-C”) experiments probe the spatial organization of chromosomes on a genome-wide scale to an extent that was previously unattainable. These experiments report on contacting loci and thus provide information about the three-dimensional structure of the genome. Unfortunately, the data are noisy and do not determine the structure uniquely. There is also little quantitative prior knowledge about the large-scale organization of chromosomes. Here, we address these challenges by developing a Bayesian statistical approach that combines a minimalist polymer model with chromosome size measurements and conformation capture data. Our method generates statistical ensembles of chromosome structures from extremely sparse single-cell Hi-C data. We remove potential bias by learning modeling parameters from the experimental data and apply model comparison techniques to investigate which among a set of alternative models is most supported by the Hi-C data. Our method also allows for modeling with ambiguous contact data obtained on polyploid chromosomes, which is an important step towards three-dimensional modeling of whole genomes.

## Introduction

The rapid development of chromosome conformation capture techniques such as 3C [1], chromosome conformation capture-on-chip [2], the closely related circular chromosome conformation capture [3] (both named 4C) and 5C [4] culminated in a genome-wide variant, Hi-C [5], which provides all-against-all contact information. Hi-C experiments confirmed previously established hallmarks of genome organization including the existence of chromosome territories [5, 6] and led to important new findings such as the partitioning of chromosomes into alternating active and passive, megabase-sized compartments [5, 7] and in non-tissue-specific topologically associating domains (TADs) on the sub-megabase scale [8–10].

Chromosome conformation capture experiments typically analyze populations of millions of cells, thereby only providing a population-averaged view. Recently, however, Nagano *et al.* [11] pioneered Hi-C on single cells by executing all of the steps of the original Hi-C protocol within permeabilized cells and selecting individual cells for further analysis. Although the single-cell Hi-C approach provided only very sparse contact data, the structural information was sufficient to reveal unprecedented insights into genome organization including a high cell-to-cell variability of interdomain and *trans*-chromosomal contacts as well as the persistence of TADs across single cells.

Many structural insights such as the existence of TADs or the scaling behavior of contact probabilities with genomic distance can be found by analyzing genome-wide contact matrices. Nevertheless, it seems attractive to obtain a more direct view of the 3D architecture of genomes by structural modeling based on the experimental contact information. To compute representative 3D structures of genomes, various approaches have been explored.

There is a growing array of computational methods for calculating consensus structures from population Hi-C data. Typically, these methods first derive distances from the experimental contact frequencies by using different heuristics. In the early work by Duan *et al.* [12], a model of the yeast genome was computed based on data from a 4C-related experiment. Bau *et al.* [13] mapped inverse log Z-scores from 5C data to distances and used the Integrated Modeling Platform (IMP) [14] to compute structural models. PASTIS [15] addresses chromosome structure determination by means of maximum likelihood, whereas ChromSDE [16] relies on semi-definite programming. Along with the structure, both PASTIS and ChromSDE optimize an additional free parameter, which is used to translate contact counts into distances. Trieu *et al.* [17] used an optimization-based approach, but modeled contact counts explicitly. Also Zhang *et al.* [18] avoided the conversion of contact counts to distances, but instead of a consenus structure, they obtain structure ensembles by simulating from an approximate energy landscape for chromosomes.

Two major challenges complicate the adaptation of methods for chromsome structure inference from population Hi-C to single-cell data. First, single-cell Hi-C measures only the formation of a contact rather than contact frequencies. Second, only a small subset of all chromosomal contacts is measured such that the contact information is very sparse. Therefore, specialized methods for the analysis of single-cell Hi-C contacts need to be developed. Multidimensional scaling (MDS) is a popular method to obtain three-dimensional structures from incomplete and noisy distance information and was already used in the first publication on chromosome conformation capture [1]. A major limitation of MDS is that with dwindling number of data serious artifacts are introduced, which eventually leads to a complete break-down of the procedure. Shortest-path reconstruction in 3D (ShRec3D) [19] and an approach employing manifold-based optimization (MBO) [20] are two recent variants of MDS that aim to overcome these challenges for single-cell Hi-C data. Both methods define a contact distance between loci that show a contact in the Hi-C experiment and introduce a similar distance between neighboring loci along the chromatin fiber. The missing entries of the distance matrix are imputed by shortest-path distances, which are computed for a graph derived from the experimental contacts and the fiber connectivity. ShRec3D applies MDS directly to the completed distance matrix, whereas Paulsen *et al.* downweigh the shortest-path distances and utilize optimization techniques on matrix manifolds [21]. Nagano *et al.* [11] used restraint-based modeling to obtain structures of the X chromosome from their single-cell data. They derived distances from the contact data and combined the restraint energy with a simple polymer model. To find chromosome structures that fit the restraints, they used simulated annealing (SA) combined with molecular dynamics.

However, the application of optimization approaches such as SA or MDS to chromosome structure determination suffers from the same conceptual problems described by Rieping *et al.* [22] in the context of protein structure calculations from NMR data. First, the scoring function typically involves model parameters that are unknown and set to *ad hoc* values. An example is provided by the weighting factors that define the “strictness” of the restraints [23]. Second, structure ensembles generated by minimization approaches lack a sound statistical foundation. These ensembles are computed by running multiple minimizations from randomly varying initial structures. Although this practice seems plausible, the variability of the ensemble is not a valid “structural error bar”, because it does not only reflect the quality and amount of the data, but also the power of the optimization procedure, to mention but one reason. Third, minimization approaches fail to clearly separate model parameters from algorithmic parameters, again blurring the meaning of the structure ensemble.

To address these issues, Rieping *et al.* [22] introduced Inferential Structure Determination (ISD) as an unbiased and parameter-free alternative to minimization approaches to biomolecular structure determination. ISD is a Bayesian probabilistic framework that views biomolecular structure determination as an inference problem. At the core of the ISD approach is a probability distribution over conformational space representing and combining both noisy and possibly incomplete data as well as prior knowledge about the unknown structure. Several Bayesian approaches have been developed to model the structure of chromosomes based on ensemble Hi-C data [24, 25]. These methods try to infer a consensus structure of the population data, which is only of limited use. Moreover these approaches have not fully benefited from the Bayesian approach to chromosome structure inference. Wang *et al.* [26] calculate structural ensembles using a Bayesian approach, but only optimize the posterior probability and do not apply the full Bayesian inference machinery, which allows for the quantification of parameter uncertainties and the comparison of alternative models.

Here we report on the application of ISD to infer statistically well-defined ensembles of chromosome structures from single-cell Hi-C data. We show that Markov chain Monte Carlo (MCMC) sampling allows us to compute diverse ensembles of coarse-grained chromosome conformations that reflect the sparsity of single-cell Hi-C contacts. MCMC techniques and the flexibility of our Bayesian approach also allow us to compare different models of the chromatin fiber as well as alternative models for Hi-C contacts. We use the conformational ensembles to map epigenetic marks into three-dimensional space. Furthermore, we demonstrate that ISD outperforms alternative methods on simulated data. Finally, we show how to extend the approach to diploid chromosomes and infer the structures of two chromosome copies simultaneously.

## Results

### Chromosome models

We model the chromatin fiber with a beads-on-a-string representation. Owing to the sparsity of single-cell Hi-C contacts, we use a highly coarse-grained model in which every bead represents 500 kb of chromatin and has a radius of approximately 215 nm. Beads are connected such that they form a linear chain. The connectivity is enforced by a harmonic backbone potential, which penalizes distances between consecutive beads as soon as they exceed the bead diameter *a*. Beads are soft and allowed to overlap to some extent. We investigated two volume exclusion terms: a purely repulsive potential under which two beads that are closer than their diameter repel each other, and a Lennard-Jones potential with repulsive and attractive contributions. The sum of the backbone and nonbonded potential are part of the prior distribution (see Materials and Methods for details).

**Fig 1:**
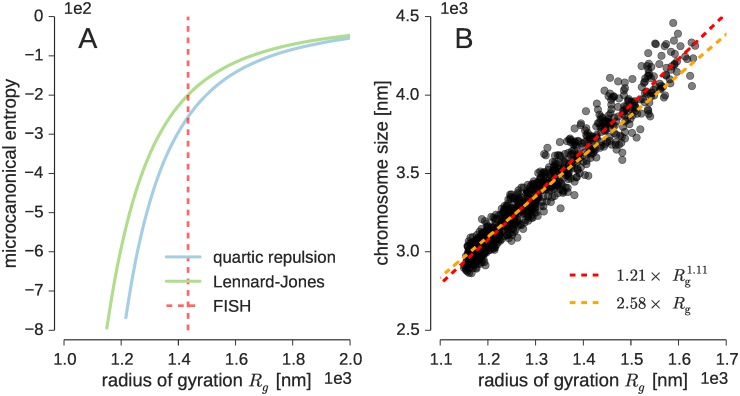
Overall properties of the chromosome model. **(A)** Density of states (microcanonical entropy) of the radius of gyration for both excluded volume potentials. **(B)** Relation between the radius of gyration and the size of the X chromosome.

We first studied the properties of our model for the single-copy X chromosome of male mouse, which measures 166 Mb in length, and which we represent using 333 beads. We generated structures from the prior distribution and reconstructed the distribution of the radius of gyration *R*_g_ as a measure for the compactness of the fiber. Because there are no or only weak attractive interactions between the beads, the vast majority of structures generated from the prior showed an extended conformation. Fig 1A displays the density of states (or equivalently the microcanonical entropy) as a function of the radius of gyration. The density of states counts how many chromosome conformations map to a particular *R*_g_ value. There is a strong entropic force that pushes the fiber into an extended state characterized by a large radius of gyration. For the repulsive excluded volume term we found *R*_g_ = 11.2 ± 1.1 μm; for the Lennard-Jones potential we have *R*_g_ = 8.6 ± 1.3 μm. Due to the attractive contribution in the excluded volume term, the Lennard-Jones potential shows a higher preference for compact structures. Yet for both potentials, the fraction of compact structures with *R*_g_ smaller than 2 μm, say, is vanishingly small: 1.8 × 10^−24^ for the quartic repulsion potential and 7.8 × 10^−21^ for the Lennard-Jones term.

### Incorporation of FISH measurements

The preference of the prior probability for extended structures with large average radii of gyration is incompatible with fact that chromosomes localize in chromosome territories, i.e. relatively compact subcompartments of the nucleus [6]. To good approximation, the size of the chromosome is a function of the radius of gyration. Empirically, we found that $1.21\times R_{g}^{\;1.11}$ gives a good estimate of the size of the X chromosome for relatively compact structures (Fig 1B); a simpler, yet precise enough relation is 2.58 × *R*_g_. Using this approximation, the experimental chromosome size measurement of 3.7 ± 0.3 μm obtained with X-chromosome paint FISH [11] corresponds roughly to *R*_g_ ≈ 1.43 ± 0.12 μm. Therefore, the average radii of gyration reported above are an order of magnitude too large compared to the experimental finding.

To incorporate the information from FISH into our probabilistic chromosome model and thereby inform the prior probability about the expected chromosome size, we assume a Gaussian error model for the chromosome size measurements. Based on our approximate relation between chromosome size and *R*_g_, this term corresponds to a harmonic radius of gyration restraint with an experimental *R*_g_ value of 1.43 ± 0.12 μm.

### Inferential structure determination of the X chromosome from single-cell Hi-C contacts

By using probability calculus, it is possible to combine single-cell Hi-C contacts with the FISH data and our chromosome models (see Materials and Methods). Nagano *et al.* [11] analyzed Th1 cells of male mouse and selected ten cells that passed various quality criteria. We first focused on the most promising data set from cell 1 showing 616 X-chromosomal *cis*-contacts, which represents the highest number of contacts among all ten cells. We mapped these contacts onto the 500 kb beads; the removal of intra-bead contacts (“self-contacts”) resulted in 438 contacts, out of which 399 were unique (some contacts mapped onto identical pairs of beads).

We used a logistic model to quantify the probability that a *cis*-chromosomal contact is observed in a Hi-C experiment. The logistic function is a smooth version of a step function and has probability close to one if the contact is formed in the model structure of the fiber, and vanishes if the beads are too far apart. Consistent with other approaches [27, 28], we chose a distance cutoff *d*_c_ = 1.5 × *a* ≈ 650 nm to decide whether two beads *i* and *j* are in contact. The smoothness of the logistic function was chosen such that a distance violation of 2.5% of the contact distance *d*_c_ has a probability smaller than 10^−6^.

We generated ensembles of X-chromosome structures for both excluded volume potentials, both without and with the additional model for FISH data introduced in the previous section. To sample chromosome conformations we used Hamiltonian Monte Carlo (HMC) [29], a stochastic variant of molecular dynamics. We started the HMC simulation from a fully extended X-chromosome structure. To ensure correct conformational sampling, we used replica exchange Monte Carlo [30, 31] which runs multiple HMC simulations at different temperatures in parallel and exchanges conformations between the different simulations (see Materials and Methods). Replica exchange simulations are among the most powerful Monte Carlo methods to simulate complex probability distributions but computationally demanding. It is also possible to generate X-chromosome conformations that satisfy the experimental contacts by only running HMC at a fixed temperature. The HMC sampler rapidly finds a compact structure that fits the contact data very well without producing significant violations. Nevertheless, the following results were obtained by running replica exchange simulations.

**Fig 2:**
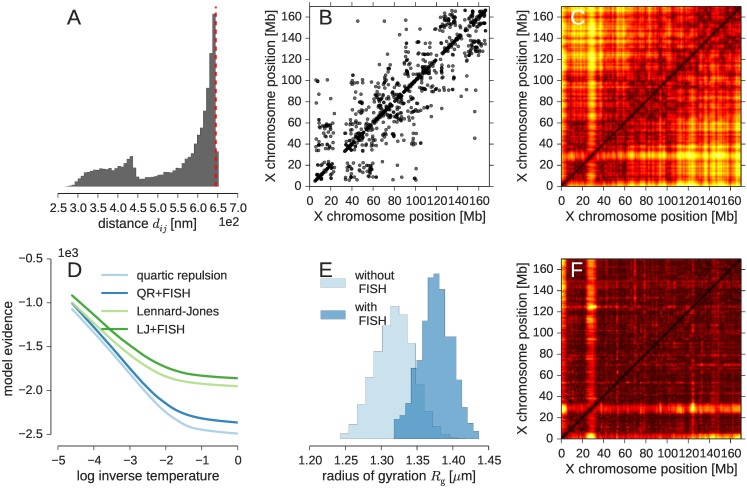
Inferential structure determination of the mouse X chromosome from single-cell data. **(A)** Pooled histogram of all distances involved in an experimentally observed contact. The left peak corresponds to experimental contacts between consecutive beads that are neighbors in the chromosome fiber. The red dashed line marks the contact distance *d*_c_. **(B)** *Cis*-chromosomal contacts from single-cell Hi-C and **(C)** distance matrix obtained with the Lennard-Jones potential. **(D)** Model evidence for both excluded volume potentials without and with FISH data shown as a function of the replica temperature. **(E)** Distribution of the radius of gyration for the Lennard-Jones prior without and with FISH data. **(F)** Matrix of standard deviations of pairwise distances reflecting the spread of the sampled X-chromosome conformations. Shown is the result for the Lennard-Jones potential with additional FISH data. The color palette ranges from black to yellow indicating small to large standard deviations.

As a first validation of our inference approach we studied whether all experimental contacts can be satisfied in the 3D model of the X chromosome. Fig 2A shows that this is indeed the case. For all four prior distributions the number of violations fluctuates about a small percentage of less than 3% (see also S1 Fig). By increasing the steepness of the logistic function, we could decrease this number to exactly zero.

As a further validation we analyzed the average pairwise distance matrix computed from the sampled X-chromosome structures. Fig 2B and 2C compare the experimental contacts with the average distance matrix. We observe that loci with a high number of *cis*-chromosomal contacts correspond to patches of small pairwise distances in the distance matrices. Using a diagonal permutation test, we found that the average distance matrices based on the different prior probabilities agree to a large extent, which underlines the fact that the prior does not have a strong influence on the average properties of the structure ensemble. The correlation of the distance matrices ranges between 93% and 95% (see S1 Fig for details).

Nevertheless, we can use Bayesian model comparison to study whether the data show any preference for one of the prior probabilities. To do so, we estimated the model evidence (also known as marginal likelihood) from the MCMC simulations. The model evidence quantifies how likely a probabilistic model is in the light of the data. Fig 2D shows that the contact data prefer the Lennard-Jones term over the quartic repulsion term. The incorporation of the information from FISH raises the model evidence further (see S1 Table).

Fig 2E shows the radii of gyration obtained without and with FISH data when using the Lennard-Jones potential. The structures generated with the *cis*-chromosomal contacts only, without additional FISH data are already quite compact with an average *R*_g_ of 1.32 μm. Due to the strong forces exerted by the logistic contact restraints, the ensemble is slightly more compact than suggested by the FISH data. When incorporating the FISH term, the average *R*_g_ shifts towards larger values with an average of 1.38 μm, corresponding to a chromosome size of ∼3.6 μm. The FISH data do not compromise the fit with the contact restraints: the number of violations does not change upon incorporation of the *R*_g_ model (see S1 Fig). However, because the additional radius of gyration term helps to focus the conformational sampling on reasonably compact chromosome structures, the model evidence of the FISH based posterior is higher than without FISH data.

By looking at the variance of the pairwise distances in the structure ensembles (Fig 2F and S1 Fig), we find that the regions at the start of the X chromosome and around the centromere show the highest degree of conformational diversity. It is unclear, however, if this increased variability reflects true conformational fluctuations or simply the fact that these regions are unmappable. Nonetheless, we also observe an overall rise in the distance fluctuations towards the telomeric region, which might indicate that this part is indeed more dynamic.

### Modeling single-cell Hi-C data as distance measurements

Many approaches to infer chromosome conformation from Hi-C data resort to modeling based on distance restraints. To this end, pairwise distances need to be derived from the experimental contact information. For example, the contact frequencies measured in ensemble Hi-C experiments were converted to distances by assuming a power law that relates the contact probability to the inter-bead distance, which is motivated by results from polymer physics (see e.g. [15]). In case of single-cell data, this is more challenging because only single contacts are observed and not contact frequencies.

To interpret the observation of a single-cell Hi-C contact as a distance measurement, we introduce an unknown distance *δ* between two loci that will be crosslinked. For the unknown chromosome conformation *X* it should therefore hold that *δ* ≈ *d*_*ij*_(*X*) where *d*_*ij*_(*X*) is the model distance between beads *i* and *j* representing both loci. Because *δ* is unknown, we estimate this model parameter simultaneously with the chromosome structure. In contrast, Nagano *et al.* [11] used $a/n_{ij}^{2}$ as experimental distance where *a* denotes the bead diameter and *n*_*ij*_ counts how often beads *i* and *j* form a contact after mapping the high-resolution contacts onto the coarse-grained representation of the fiber. At 500 kb resolution *n*_*ij*_ ranges from 1 to 3. In our approach, the repeated occurrence of a contact (*n*_*ij*_ > 1) does not lead to a shortening of the contact distance, but rather to an enforcement of the distance restraint, which is duplicated *n*_*ij*_ times.

Due to experimental errors and shortcomings of our model, we have to account for discrepancies between the unknown experimental distance and the model distances. This is achieved by introducing a probabilistic model for the distribution of the discrepancy between *δ* and *d*_*ij*_(*X*). We studied two error distributions: The first assumes a Gaussian shape with a flat plateau for distances between *δ* ± 0.2 × *a* in accordance with the approach by Nagano *et al.* [11]. The second model is a lognormal distribution [32]. Both error models depend on an additional unknown error parameter *σ*, which reflects how well the experimental distance agrees with the model distance. The inverse variance *w* = 1/*σ*^2^ can be interpreted as the weight of the distance restraint potential [23]. We set *w* to relatively large values to reflect our assumption that all observed contacts are correct (which we also assumed in the logistic contact model). We used *w* = 100 for the Gaussian with flat plateau and *w* = 500 for the lognormal model, because it has a softer shape than the harmonic restraint resulting from the Gaussian model.

**Fig 3:**
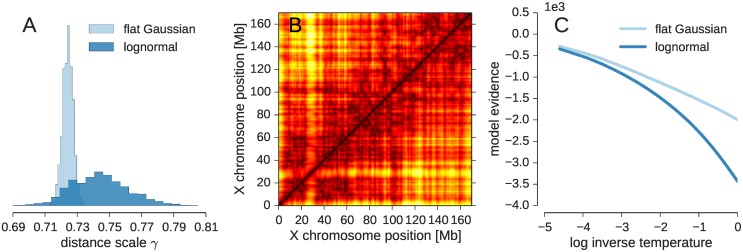
Distance based modeling of the X chromosome. **(A)** Histogram of sampled distance scale estimates γ. **(B)** Average distance matrix for the Gaussian error model with a flat plateau (upper diagonal) and for the lognormal model (lower diagonal). **(C)** Log evidence log Pr(*D*|*I*) for different data weighting parameters λ (the inverse temperature λ is varied from 10^−4^ to 1 in a replica exchange simulation to facilitate sampling of the posterior distribution).

To estimate the experimental distance using ISD, we rewrite *δ* = *a*/γ where γ > 0 is an unknown scaling parameter such that ideally *a* = *γd*_*ij*_(*X*). Fig 3A shows histograms of the estimated distance scale γ. The distance scale γ attains similar values for both error models. The average values are 0.724 ± 0.003 for the Gaussian with a flat plateau and γ = 0.744 ± 0.018 for the lognormal model. These values translate into an estimated inter-locus distance of ∼1.4 × *a*, which is comparable to the contact distance *d*_c_ assumed in the logistic contact model. The distances involved in an experimental contact (shown in Fig 2A for the contact model) are less restrained in the distance-based models (see S2 Fig). Nevertheless the agreement between the structure ensembles generated with both distance-based models is fairly high. The correlation between the average distance matrices is 95% (Fig 3B), and both ensembles also agree well with the ensemble based on the logistic contact model (with a correlation coefficient of 96% between the average distances matrices generated by each of the distance-based models and the contact model).

Using Bayesian model comparison, we can also answer which of the two error models, Gaussian with a flat plateau or lognormal model, is preferred by the experimental data. Fig 3C shows the model evidence Pr(*D*|*I*) as a function of the replica temperature parameter λ. The values for λ = 1 indicate that the data tell us to prefer the Gaussian model with a flat plateau over the lognormal error model.

By running calculations in which we varied the error parameter *σ*, we found that the exact value of the distance scale γ depends on the assumption that we make about the reliability of the Hi-C data to some extent (see S3 Fig). However, for reasonably small *σ*/large *w* this dependence is less strong and the distance scale γ reaches a plateau. Bayesian inference also allows us to estimate both model parameters, γ and *σ*, simultaneously, but this results in rather broad ensembles that do not adopt a well-defined structure (see S1 Appendix). The failure to generate well-defined structure ensembles, if *w* is allowed to vary is due to the sparsity of the data: The intrinsic tendency of the chromatin fiber to adopt a disordered state is stronger than the forces exert by the distance restraints with variable weight. Only with a large enough weight it is possible to counter-balance the entropic forces. Another remedy is to improve the model of the chromatin fiber [33], but this is beyond the scope of this article.

Our results show that it is possible, in principle, to model single-cell Hi-C contacts as distance measurements. However, we will use the logistic contact model in the remainder of this article, because single-cell Hi-C observes binary contacts rather than continuous distances.

### Detailed analysis of the X-chromosome structure ensemble at 500 kb resolution

**Fig 4:**
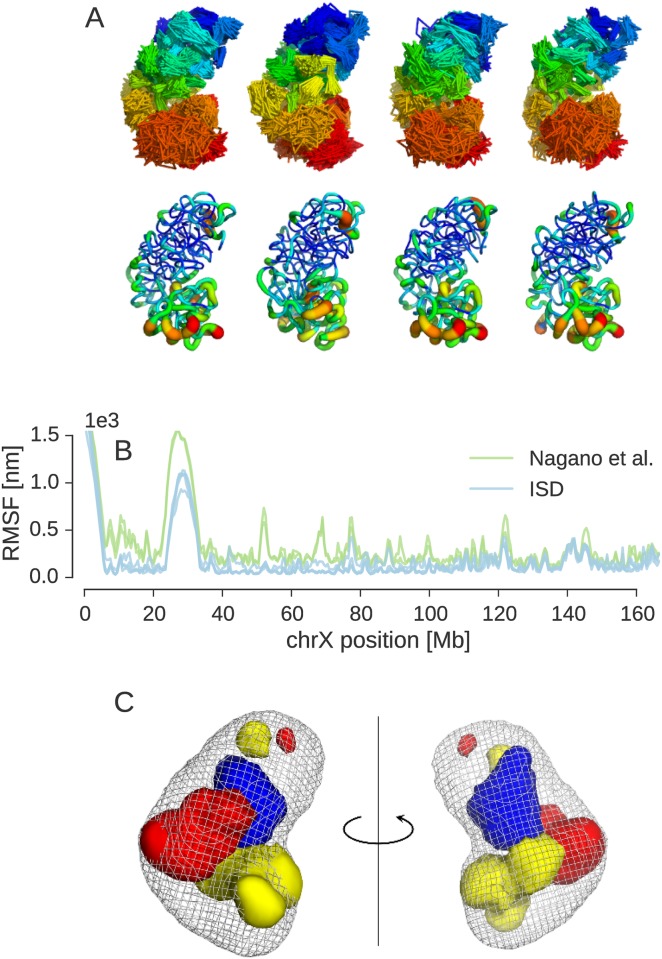
X-chromosome structure ensemble at 500 kb resolution. **(A)** Four principal conformers representing the posterior ensemble. Top: Trace plots showing all members of each cluster. The color palette indicates chromosome position ranging from centromere (blue) to telomere (red). Bottom: “Sausage representation” with the tube thickness indicating the local precision of bead positions. Unmappable beads 1–10 (1–5 Mb) and 48–66 (24–33 Mb) have been omitted for clarity. **(B)** Root mean square fluctuations (RMSF) within each cluster of the ISD ensemble and within clusters of the ensemble from the original single-cell Hi-C publication. **(C)** Three-dimensional feature maps for the first structural cluster. Regions involved in *trans*-chromosomal contacts are shown as red density. Lamin-B1 enriched regions are shown in yellow. H3K4me3 enriched regions are shown as blue volume. The overall shape of the cluster is shown in mesh representation. The orientation of the maps shown on the left is identical to the orientation of the ensemble shown in panel **(A)**. The maps shown on the right are rotated by 180° about a vertical axis.

We now take a closer look at the ensembles generated with the ISD approach and compare them to the published ensemble by Nagano *et al.* [11]. The structure of the X chromosome adopts a bipartite conformation formed by two super-domains that approximately span the centromeric half (∼1–100 Mb) and the telomeric half (∼100–166 Mb) of the chromatin fiber. This large-scale domain structure is readily apparent from both the experimental contacts and the average distance matrix (Fig 2C) and becomes immediately visible in an explicit representation of the structure ensemble (Fig 4A).

Cluster analysis reveals that the ISD ensemble comprises multiple principal conformations about which the structures fluctuate. Closer inspection shows that the cluster centers are partial mirror images of each other. None of the likelihood and prior factors contributing to the posterior distribution distinguishes between a particular bead configuration and its mirror image, because all factors depend on distances only. Moreover, since there are only few contacts between the super-domains, each super-domain can show two conformations which are mirror images of each other. This results in at least four possible chromosome conformations which all achieve a similar goodness of fit of the Hi-C contacts.

Our cluster analysis finds that the eight most dominating structural clusters produced by ISD cover ∼90% of all states sampled from the posterior (see S4 Fig for further details). The four most highly populated clusters are shown in Fig 4A. These are approximate mirror images of each other. We applied the same type of cluster analysis also to an ensemble of 200 X-chromosome structures computed by Nagano *et al.* [11] (see S5 Fig). We found similar structural clusters that are partial mirror images of each other and have corresponding structural clusters in our ensemble (details given in Supplementary Information). However, overall the ISD ensemble seems to be more diverse, showing more clusters than the ensemble by Nagano *et al.*

Visual inspection of the structural clusters suggests that the overall variability in the ISD ensemble is quite high and comparable to the fluctuations in the ensemble by Nagano *et al.* But each cluster of the ISD ensemble appears to be slightly better defined than the clusters in the ensemble by Nagano *et al.* This might be due to the more exhaustive sampling achieved by our Monte Carlo algorithm, the attractive contributions in the excluded volume term and the fact that we model Hi-C measurements as contacts with a steep sigmoidal contact probability rather than distance restraints.

For each cluster, we studied the local variability of the beads by using standard techniques for the analysis of NMR structure ensembles. We estimated the local precision of the bead positions by the root mean square fluctuation (RMSF) after superposition of the cluster members onto the cluster center. Fig 4B shows RMSF curves for both ensembles. The RMSF curves from the four major clusters of the ISD ensemble correlate almost perfectly, the same is true for the clusters of the previously published X chromosome ensemble. We also find a high agreement between the RMSF fluctuations in the ISD clusters and the fluctuations within the clusters of the ensemble by Nagano *et al*. The average Pearson correlation coefficient of the RMSF profiles is 91%, showing that the ensembles obtained with both approaches agree strongly in their conformational heterogeneity. Together with the high correlation of 85% between the average distance matrices of the ISD ensemble and the ensemble by Nagano *et al.* (see S2 Table), these findings indicate that both ensembles show many similar properties. A tube representation of the local variability of the four principal conformers is shown in panel 4A. Again, we find a higher conformational diversity towards the telemore, which was already apparent from the standard deviation of pairwise bead distances (Fig 2F).

We also ran ISD simulations on contact data from 5 additional Th1 cells. The average distance matrices indicate that the ensembles are significantly different, indicating the cell-to-cell variability of chromosome conformations found by Nagano *et al.* [11] (see S6 and S7 Figs). A comparison of the ensembles obtained with data from cell 1 to cell 6 is reported in S2 Table. This comparison shows that there is an overall agreement between the X-chromosome conformations obtained with the ISD approach and the restraint-based modeling approach by Nagano *et al.* also for the data sets from other cells.

By comparing the average distance matrices (S6 Fig), we conclude that chromosome structures from different cells share some common features, such as the partitioning into more or less well-defined domains, which appear as blocks of small distances along the diagonal. The size and location of these domains can differ significantly from cell to cell. While in cells 1, 4, 5 and 6, a well-defined telomeric domain is visible as a block between ∼130 − 166 Mb, it is much less pronounced in cells 2 and 3. This difference is also evident in the structural models (S7 Fig), in which the telomeric domain appears as a separated structural domain (colored in red). In the models for cell 4, the telomeric domain is on average ∼10 Mb shorter than in the models for cell 1, 5 and 6. The six different models also exhibit considerable differences in the spatial proximity of loci that are far apart in sequence. An example involves the centromeric region on the lower left of the average distance matrices. In the ensemble representing the X chromosome in cell 6, this region is spatially close to many loci that are up to ∼100 Mb distant in sequence, while in cell 5, this region shows significantly larger distances to most other loci. S7 Fig confirms this by showing that the blue and cyan parts of the X chromosome structure are much more exposed in cell 5 than in cell 6. Taken together, a picture of highly variable, presumably stochastic chromosome organization emerges, in which conformations nevertheless share large-scale properties across different cells.

A problem with current Hi-C based chromosome modeling is that it is difficult to validate the calculated structures. However, there are some independent sources of information, not used during modeling, that should be consistent with a meaningful structure ensemble. One is the information provided by population Hi-C. Although population Hi-C looks at a large pool of cells, the information about the *absence* of contacts should also hold for the chromosome structure based on single-cell data. As can be seen from S8 Fig by comparing the contact probabilities derived from the ISD ensemble with population Hi-C data, the ISD ensemble based on single-cell data indeed avoids contacts between loci that have a low contact probability in the population Hi-C map. Another validation is provided by the location of beads involved in *trans*-chromosomal contacts. Fig 4C and S9A Fig show that beads which are engaged in contacts with loci on other chromosomes tend to accumulate on the periphery of the structure ensemble, which is indirect evidence in support of our chromosome ensembles.

Based on the inferred structure ensemble it becomes possible to generate three–dimensional maps of genomic and epigenetic features and to correlate the features spatially. Fig 4C shows a volume representation of chromosomal regions that are enriched in H3K4me3 and lamin-B1 associated domains in the first structural cluster. Loci that are enriched in these epigenetic marks tend to aggregate in three-dimensional space. The lamin associated domains as well as H3K4me3-enriched regions both show a tendency to locate in the periphery of the X chromosome, where they occupy distinct regions. In accord with previous findings by Nagano *et al.*, we also find that H3K4me3 is enriched in some parts of the interior of the X chromosome (see S9C Fig).

### Modeling X chromosome at 50 kb resolution

Due to the sparsity of the chromosomal contacts, we have used a very coarse-grained representation of the chromatin fiber. At this resolution, we can only study large-scale chromosomal organization. Higher resolution representations are typically needed to gain biologically relevant insights into 3D chromosome organization. We therefore also applied the ISD approach using a ten-fold higher resolved chromatin fiber. Each bead now represents 50 kb of chromatin, thereby matching the finest resolution used by Nagano *et al.* [11]. At this resolution the radius of a bead amounts to ∼100 nm.

At 50 kb resolution, we represent the X chromosome with 3330 spherical beads. We generated structure ensembles based on the Lennard-Jones volume exclusion term and the additional FISH restraint. We modeled the intra-chromosomal contacts with the logistic model; in contrast to Nagano *et al.* no additional “anti-contact” restraints from the ensemble Hi-C matrix were introduced.

To compare the overall properties of the structure ensembles generated at 500 kb and 50 kb resolution, we downsampled the distance matrices from the 50 kb models to match the resolution of the coarse-grained models. Downsampling was achieved by averaging 10 × 10 patches of the average distance matrix at 50 kb resolution. The downsampled average distance matrix and the average distance matrix of the low-resolution model show a correlation of 86%, indicating that the overall shape of the X chromosome at both levels of resolution is similar (see also S10 Fig).

**Fig 5:**
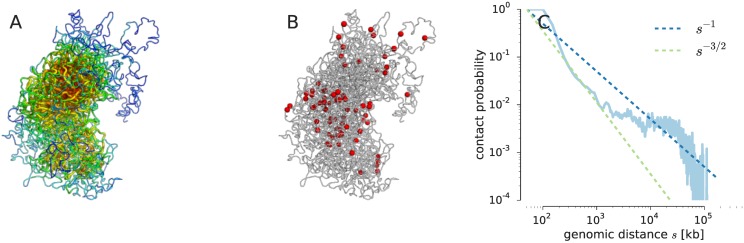
X-chromosome structure at 50 kb resolution. **(A)** Representative structure of the X chromosome at 50 kb resolution where the tube thickness and color encodes the local packing density (dense packing: red, thick; low packing: blue, thin). **(B)** Representative structure of the X chromosome with beads involved in *trans*-chromosomal contacts highlighted as red spheres. **(C)** Contact probability between beads in the ISD ensemble as a function of the genomic distance *s*. Predictions made by various polymer models are shown as dashed lines: *s*^−3/2^ ideal chain/equilibrium globule, *s*^−1^ fractal globule.

Fig 5A shows a representative conformation from the structure ensemble generated at 50 kb resolution. As with the models obtained at 500 kb resolution, the X chromosome adopts a bipartite conformation where two major domains corresponding to the centromeric and telomeric regions adopt a slightly kinked conformation. Moreover, the centromeric superdomain appears to be more densely packed. *Trans*-chromosomal contacts and epigenetic features show a similar distribution in the high-resolution ensemble in comparison with the chromosome ensemble at 500 kb resolution (see Fig 5B).

Fig 5C shows the contact frequency between beads in the ISD ensemble as a function of the genomic distance *s* (separation between beads along the fiber). In the region from 200 kb to 2000 kb genomic distance, the contact frequency shows random coil behavior. It has been argued on the basis of ensemble Hi-C data [5, 34] that chromosomes adopt a fractal globule packing. The fractal globule shows *s*^−1^ dependence of the contact frequency, in contrast to the equilibrium globule which is expected to show a scaling behavior of *s*^−3/2^ until it reaches a constant value. Our 50 kb ensemble shows a mixed packing supporting a more complicated packing than the fractal globule also in single cells.

### Probabilistic inference of diploid chromosome structure

**Fig 6:**
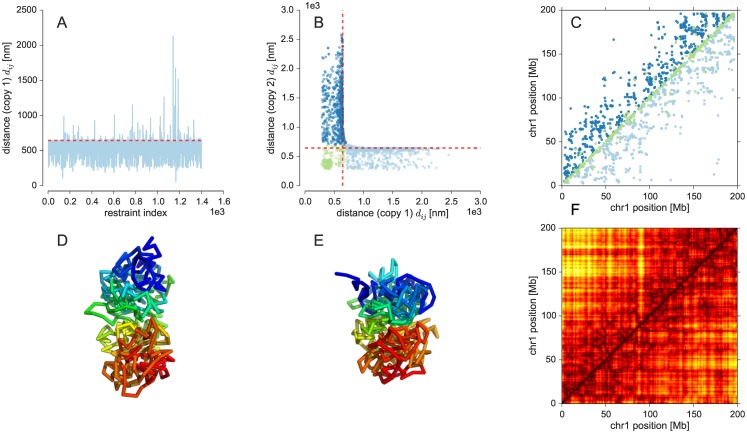
Inference of chromosome 1 structure. **(A)** Distance violations in a probabilistic calculation using a single copy of chromosome 1. The contact distance is shown as dashed red line. **(B)** Distances of contacting beads in copy 1 against distances in copy 2. **(C)** Experimental contacts classified into restraints formed in both copies (green) and contacts seen only in one of the two copies (dark blue: copy 1, light blue: copy 2). **(D, E)** Average structures of both copies of chromosome 1. **(F)** Average distance matrix with upper half showing the first copy of chromosome 1 and the lower half showing the second copy.

Because Th1 cells of male mice are diploid, most *cis*-chromosomal contacts of the single-cell Hi-C data from Nagano *et al.* show contacts involving two-copy chromosomes. The difficulty with these data is that we do not know whether a particular contact is formed in the first or the second copy of the chromosome. Therefore, we have to disentangle the contacts to use them for structural modeling. A naive approach to achieve this kind of demixing is to fit the contacts with a single chromosome structure and consider the violated restraints as contacts specifying the structure of the second copy. This approach is problematic because the first structure will try to explain as many contacts as possible, which results in a strong strain. Because the model of the fiber is very generic and flexible, it will be possible to fit a large fraction of all contacts with a single structure. This is shown in Fig 6A where we used a single structure to compute models from the single-cell *cis*-contacts of chromosome 1. Only a small fraction of less than 10% of all 1662 experimental contacts is strongly violated in the single-copy ensemble. It is hard to imagine that this small fraction of contacts is sufficient to determine a meaningful structure of the second copy. Furthermore, the number of restraints per copy would be highly unbalanced, for which there is no plausible physical reason.

A reason for the failure of the naive approach is that it tries to assign contacts to either of the two copies similar to an *exclusive-or* operation. The more appropriate operation would be a logical *or*: A contact is either formed in both chromosome copies or in only one of the two copies. To implement this approach efficiently, we can again benefit from concepts developed in NMR structure calculation. Ambiguous NMR crosspeaks showing inter-proton contacts are routinely modeled using ambiguous distance restraints (ADRs) [35]. The reason is that due to the chemical shift degeneracy a peak can often be explained by multiple alternative contacts. Which of the alternatives is the correct one, is not known at the beginning of the structure calculation. An ADR combines all alternative distances into a single average distance that has to match the experimental distance. The trick of ADRs is to average the distances not arithmetically, but by summing the inverse sixth powers of the distances followed by taking the inverse sixth root of this sum (*r*^−6^ averaging). In case of NMR structure calculation, this type of averaging can be motivated by the physical nature of the NMR signal. But we can also use it in different applications to implement some kind of *or* operation [36]: Due to the strong decay of the inverse sixth power large distances contribute only very weakly to the ADR. Therefore all configurations that could have led to the observation of the contact (contact formed in only one of the two copies and contact formed in both copies), approximately result in the same average distance.

To model two-copy chromosomes with ISD, we use two independent chromatin fibers and describe the observation of Hi-C contacts using a logistic model. However, in contrast to the single-copy approach, the logistic function is evaluated for the ADR computed by *r*^−6^ averaging of the corresponding distance in each copy. We only introduce intra-fiber distance restraints, although contacts could in principle arise also from inter-fiber contacts between the two copies. We exclude this possibility and assume that homologous chromosomes segregate into distinct territories that have no physical contact [37]. Fig 6B plots the restrained distances in the final structure of copy 1, $d_{ij}^{\left(1\right)}$, against the distances in copy 2, $d_{ij}^{\left(2\right)}$. Since there are no distance pairs that are both significantly larger than the contact distance *d*_c_, all contacts can be satisfied in only one of the two copies or in both. There is a cluster of contacts that are found in both structures ($d_{ij}^{\left(1\right)}<d_{c}$ and $d_{ij}^{\left(2\right)}<d_{c}$). Other contacts are only formed in one of the two copies (copy 1: $d_{ij}^{\left(1\right)}<d_{c}$ and $d_{ij}^{\left(2\right)}>d_{c}$, copy 2: $d_{ij}^{\left(1\right)}>d_{c}$ and $d_{ij}^{\left(2\right)}<d_{c}$). A classification of the contacts into these three classes is shown in Fig 6C. Note that the fraction of contacts that is either assigned to copy 1 or 2 is quite balanced: 42% of the contacts are assigned to copy 1, 41% are assigned to copy 2; the shared contacts are mostly found close to the diagonal. Fig 6D and 6E shows the average structure for both copies of chromosome 1. Again, we observe a bipartite domain architecture where the first copy is less compact than the second copy. Another way of looking at the structural differences between the two copies are the average distance matrices shown in Fig 6F.

Our model of the two-copy chromosome 1 suggests that homologous chromosomes exhibit a degree of structural variability within the same cell that is similar to the cell-to-cell variability across different cells. With a correlation coefficient of 51%, the difference between the distance matrices of both chromosomal copies is similar to the difference between structures of the X chromosome from different cells (see S2 Table). This finding agrees with FISH images of chromosome territories showing that homologous chromosomes can adapt very different shapes (see, for example, chromosome paint images of mouse lymphocytes [38] or human fibroblasts [39]).

## Discussion

This article introduces a Bayesian probabilistic approach to infer the three-dimensional structure of chromosomes from sparse 3C contacts measured in single cells. Our approach builds on the ISD framework, which was originally developed for NMR structure determination of proteins. It is based on a posterior probability distribution over the space of chromosome conformations and model parameters. The posterior probability distribution integrates the information from single-cell Hi-C contacts and FISH measurements with a coarse-grained model of the chromatin fiber. We demonstrate the strengths of the ISD approach for single-cell Hi-C measurements on Th1 cells of male mouse [11].

Using Markov chain Monte Carlo algorithms, we can generate statistically valid structure ensembles that represent the posterior probability. The ensembles can be used to compute a local error bar for the bead positions or the distribution of other structural parameters such as the radius of gyration and indicates which chromosomal regions are well-defined by the input data. Along with the chromosome structure, ISD also estimates model parameters such as the distance between two contacting loci.

The Bayesian framework allows us to not only estimate bead positions and model parameters but also to compare alternative descriptions of the chromosome fiber. Here we compared two different volume exclusion potentials, a purely repulsive potential and a potential with both attractive and repulsive contributions, and showed that the latter is preferred by the experimental data. Our approach can also quantify the information content of individual data sets. We showed that the incorporation of FISH data helps to define the chromosome conformation. Bayesian model comparison can also be used to select the best among alternative models of the experimental contacts in a data-driven fashion. This is demonstrated here for two distance-based restraint potentials.

All of these findings are shown in detail for the single-copy X chromosome at 500 kb and 50 kb resolution as well as demonstrated on synthetic data. It is also possible to analyze contact data for diploid chromosomes. By using ambiguous distance restraints, again a concept from protein NMR, we can also infer the structures of both chromosome copies simultaneously and thereby disentangle intra-chromosomal contacts that stem from either of the two copies. These features are beyond the scope of previous methods such as restraint-based modeling [11], MDS approaches [19, 20] and a recent embedding method based on semidefinite programming [16].

There are many possibilities for future applications and extensions of ISD to chromosome conformation capture data. An important aspect will concern the implementation and testing of more elaborate models of the chromatin fiber. Here we worked with a homopolymer model according to which the fiber is composed of equally sized beads that all interact by means of a single volume exclusion potential. This model ignores many known properties of chromosomes such as the existence of hetero- and euchromatin. Nucleosome positioning and other epigenetic data could be used to define a more realistic model of the fiber. Moreover, it might be required to introduce additional prior terms that control the persistence length and local flexibility. More realistic chromosome models have been proposed such as the Strings and Binders Switch (SBS) model [40]. These models could replace our current coarse-grained prior representation. By using Bayesian model comparison, we could then quantify whether the data support a more elaborate model of the chromatin fiber. Moreover, it should be possible to estimate the optimal resolution of the fiber from the data.

Here we focused on the use of single-cell Hi-C data. It is straightforward to also incorporate contacts from ensemble Hi-C experiments into our Bayesian modeling framework. One possibility would be to model scarcely populated contacts in the ensemble Hi-C matrix as “anti-contacts”, although the absence of contacts has to be interpreted with caution [41]. ISD can also be used to generate consensus structures using contact or distance restraints from ensemble Hi-C data comparable in spirit to the approach by Trieu *et al.* [17] or other probabilistic approaches to compute consensus structures. However, these ensembles will only be of limited use, because a consensus shows the average of many structural states and can give a misleading picture of genome organization [41]. In future work, we will therefore try to generalize the ISD approach to properly treat ensemble Hi-C data.

### Conclusion

In conclusion, our work shows that ISD provides a statistically sound and viable alternative to restraint minimization or embedding approaches for single-cell Hi-C data and produces less biased and statistically valid ensembles of chromosome conformations.

## Materials and Methods

### Inferential Structure Determination

The Inferential Structure Determination (ISD) approach [22] views the determination of macromolecular structures as a problem of statistical inference and asks how likely a structure *X* is in the light of incomplete and noisy data *D* as well as prior information *I*. In the language of probability theory, the answer is provided by the probability distribution Pr(*X*|*D*, *I*) defined over the entire conformation space. ISD uses Bayes’ theorem to compute the posterior probability Pr(*X*|*D*, *I*):

$$\mathrm{Pr}\left(X|D,I\right)=\frac{\mathrm{Pr}(D|X,I)\;\mathrm{Pr}(X|I)}{\mathrm{Pr}(D|I)}$$ (1)

where Pr(*D*|*X*, *I*) is the likelihood, Pr(*X*|*I*) the prior probability and Pr(*D*|*I*) the model evidence or marginal likelihood.

The likelihood Pr(*D*|*X*, *I*) quantifies the agreement between the data and the structural model. To construct the likelihood, we typically choose a *forward model* for calculating mock data from a structure *X* and an *error model* that assesses the agreement between experimental and mock data. The forward model may involve unknown parameters such as scaling factors; the error model depends on unknown noise levels. Therefore, the 3D structure *X* is not the only unknown parameter in our inference problem. Additional model parameters *θ* need to be inferred from the data along with the conformational degrees of freedom. This is accomplished with the augmented posterior distribution

$$\mathrm{Pr}\left(X,\theta |D,I\right)=\frac{\mathrm{Pr}(D|X,\theta ,I)\;\mathrm{Pr}(X,\theta |I)}{\mathrm{Pr}(D|I)}\;.$$ (2)

After specifying the prior distributions reflecting our background knowledge (e.g. a force field for the conformational parameters or the positivity of a scaling parameter), the problem of inferring the unknown structure and additional model parameters is formally solved. Because the posterior distribution is typically of a nonstandard form and exhibits multiple modes, we have to use powerful random sampling techniques such as Markov Chain Monte Carlo (MCMC) [42] to generate representative structures and model parameters (see below).

### Chromosome models and conformational prior probabilities

**Fig 7:**
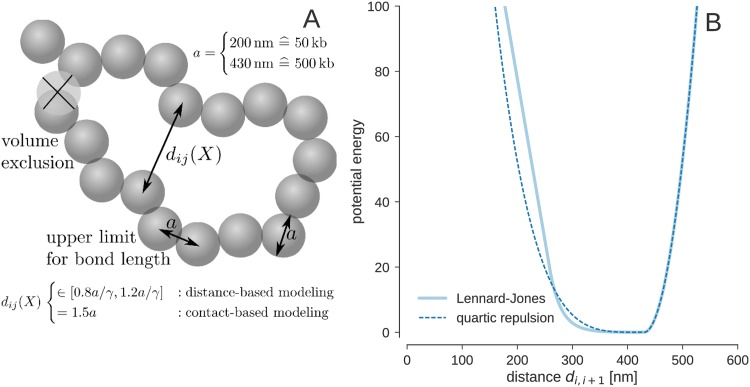
Coarse-grained chromosome model. **(A)** The chromatin fiber is composed of beads representing 500 kb of chromatin. Every bead is modeled as a spherical shape of size *a*. Configurations with overlapping beads are penalized using a nonbonded force field accounting for volume exclusion. The beads are arranged as a linear chain where the spacing between the beads is chosen such that two neighboring beads touch each other in the equilibrium configuration. **(B)** Effective potential between consecutive beads along the chromatin fiber. The dashed vertical line indicates the size of the spherical bead *a* = 430 nm.

We represent a chromosome by *N* spherical beads of diameter *a*, which determines the length scale of the model (Fig 7). A typical value for the density of a chromosome is 12 Mb/ μm^3^ [43]. Our coarse-grained model uses beads representing 500 kb of chromatin such that the volume occupied by a single bead is 1/24 μm^3^. Therefore the radius of a bead is approximately 215 nm. At a resolution of 50kb, the bead radius is approximately 100 nm.

The distance *d*_*i*, *i*+1_ between two consecutive beads *i*, *i* + 1 is restrained to an upper limit of *a* above which deviations are penalized quadratically with a force constant *k*_bb_ = 250/*a*^2^. Overlaps between two beads are penalized using an excluded volume term *E*_nb_ with force constant *k*_nb_. The conformational prior distribution defined over bead positions *X* = (*x*_1_, …, *x*_*N*_) is given by the canonical ensemble:

$$\mathrm{Pr}\left(X|I\right)\propto \exp\left\{-k_{\text{nb}}E_{\text{nb}}\left(X\right)-k_{\text{bb}}E_{\text{bb}}\left(X\right)\right\}$$ (3)

with

$$E_{\text{bb}}\left(x\right)=\sum_{i}\theta \left(d_{i,i+1}-a\right)\;{\left(d_{i,i+1}-a\right)}^{2}$$ (4)

where *I* denotes all prior assumptions, i.e. specific values for *a*, *k*_bb_, etc. Here and in Eq 5, θ(*x*) denotes the Heaviside step function; θ(*x* ≥ 0) = 1 and θ(*x* < 0) = 0.

Here we study two nonbonded force fields to account for volume exclusion effects. The first force field consists only of a repulsive term that is activated as soon as two beads come closer than their diameter *a*. A repellent force resulting from a quartic repulsion energy pushes the beads apart:

$$E_{\text{nb}}\left(X\right)=\sum_{i<j}\theta \left(a-d_{ij}\right)\;{\left(a-d_{ij}\right)}^{4}\;.$$ (5)

The strength of the force is determined by the force constant, which we set *k*_nb_ = 5/*a*^4^. The second excluded volume term is a Lennard-Jones potential with linear asymptotes:

$$E_{LJ}\left(X\right)=\sum_{i<j}\left\{\begin{array}{cc} \frac{13-14\;s^{6}}{s^{12}}-12\;\frac{1-s^{6}}{s^{13}}\frac{d_{ij}}{a} & ;\;d_{ij}<s\cdot a\\ {\left(\frac{a}{d_{ij}}\right)}^{12}-2{\left(\frac{a}{d_{ij}}\right)}^{6} & ;\;s\cdot a<d_{ij}\leq l\cdot a\\ \frac{1-2\;l^{6}}{l^{12}}\;\frac{d_{\text{cut}}-d_{ij}}{d_{\text{cut}}-l\cdot a} & ;\;l\cdot a<d_{ij}\leq d_{\text{cut}}\\ 0 & ;\;d_{ij}>d_{\text{cut}} \end{array}\right.$$ (6)

with *s* = 0.6, *l* = 1.25, *d*_cut_ = 1.375 × *a*. The effective potentials between successive beads are shown in Fig 7B.

### Probabilistic model for FISH data

FISH experiments measure the size of chromosome territories and show that the chromatin fiber adopts a compact conformation during interphase. To incorporate FISH data into our model, we first need to measure the size of a chromosome given the bead positions *X*. The squared radius of gyration is defined as:

$$R_{g}^{2}=\frac{1}{N}\sum_{n=1}^{N}{∥x_{n}-\bar{x}∥}^{2}$$ (7)

where $\bar{x}=\frac{1}{N}\sum_{n}\;x_{n}$ is the center of mass of all beads. For compact chromosome conformations we can define the size of a chromosome as the maximum extent of a bounding box circumscribing all beads. Empirically we found that the chromosome size is proportional to the radius of gyration where the proportionality factor is ∼2.58.

Let us now assume that FISH experiments result in a list of chromosome size measurements *s*_1_, …, *s*_*M*_. We model each observation using a Gaussian error model such that the probability of observing the *m*-th size measurement is:

$$p\left(s_{m}|X,I\right)=\frac{1}{\sqrt{2\pi }\sigma_{{}_{\text{FISH}}}}\exp\left\{-\frac{1}{2\sigma_{{}_{\text{FISH}}}^{2}}{\left[\;s_{m}-2.58\times R_{g}\left(X\right)\right]}^{2}\right\}$$ (8)

where *σ*_FISH_ is the error of the FISH measurements. The complete probability of all FISH measurements is:

$$\begin{array}{cc} p(s_{1},\ldots ,s_{M}|X,I) & =\prod_{m}p\left(s_{m}|X,I\right)\\ & =\frac{1}{{\left(\sqrt{2\pi }\sigma_{{}_{\text{FISH}}}\right)}^{M}}\exp\left\{-\frac{M}{2\sigma_{{}_{\text{FISH}}}^{2}}\left({\left[\;\bar{s}-2.58\times R_{g}\left(X\right)\right]}^{2}+\Delta s^{2}\right)\right\} \end{array}$$ (9)

where $\bar{s}=\frac{1}{M}\sum_{m}\;s_{m}$ is the average chromosome size and Δ*s* is the standard deviation. For the X chromosome of Th1 cells we have $\bar{s}=3.7$ μm and Δ*s* = 0.3 μm [11].

### Probabilistic models for single-cell Hi-C data

We studied three probabilistic models to measure the compatibility of a chromosome structure *X* with single-cell Hi-C data *D*. The data is a list of experimental contacts *C* between pairs of loci *i* and *j*. Two of the three models that we used to analyze the Hi-C contacts are based on distance restraints similar to the approach by Nagano *et al.* [11]. To account for inaccuracies in the data and the model, we use an error model that describes the likelihood of a mismatch between the experimental and back-calculated distances. The third model is based on sigmoidal contact probability and prefers conformations satisfying all observed contacts similar to Trieu et al. [17]. Due to coarse graining, we might observe multiple contacts between two beads; *n*_*ij*_ denotes the corresponding number of counts in the binned contact matrix. Distance / contact restraints between beads *i*, *j* with *n*_*ij*_ > 1 are duplicated *n*_*ij*_ times.

#### Distance-based modeling of single-cell Hi-C contacts

The distance-based likelihoods assume that the observation of a single-cell Hi-C contact between loci *i* and *j* can be interpreted as the observation of an unknown distance *δ*. Ideally, it should hold that *δ* = *d*_*ij*_(*X*) where *d*_*ij*_(*X*) = ‖*x*_*i*_ − *x*_*j*_‖ is the Euclidean distance between the corresponding beads. Since *δ* is not observed directly, we will treat it as an unknown model parameter.

To do this, we take the unknown distance *δ* as proportional to the bead diameter *a*, introducing a scaling factor γ via *δ* = *a*/γ. We thus assume that our single-cell experiment gives distance measurements *a* for pairs of loci (*i*, *j*) ∈ *C* and that ideally *a* = *γd*_*ij*_(*X*) for these pairs.

An error model accounts for deviations from the ideal relationship *a* = *γd*_*ij*_. These deviations are captured with a probability distribution *p*(*a*|γ, *X*, *I*) such that the complete probability of all contacts is:

$$\mathrm{Pr}\left(D|X,I\right)=\prod_{(i,j)\in C}p\left(a|\gamma ,X,I\right).$$ (10)

Our first choice for the error model is a Gaussian distribution with a flat plateau within an upper/lower limit of ±0.2 × *a* about the ideal distance *γd*_*ij*_(*X*):

$$\begin{array}{cc} p_{{}_{\text{Gauss}}}\left(a|\gamma ,X,I\right)= & \frac{1}{\sqrt{2\pi }\sigma +0.4a}\\ & \times \left\{\begin{array}{cc} \exp\left\{-\frac{1}{2\sigma^{2}}{\left[0.8a-\gamma \;d_{ij}\left(X\right)\right]}^{2}\right\} & ;\;0.8a>\gamma d_{ij}\left(X\right)\\ 1 & ;\;0.8a\leq \gamma d_{ij}\left(X\right)\leq 1.2a\\ \exp\left\{-\frac{1}{2\sigma^{2}}{\left[1.2a-\gamma d_{ij}\left(X\right)\right]}^{2}\right\} & ;\;1.2a<\gamma d_{ij}\left(X\right) \end{array}\right. \end{array}$$ (11)

The second error model is a lognormal distribution, which was introduced by Rieping *et al.* [32] to describe errors of inherently positive quantities such as distance measurements:

$$p_{{}_{\text{LN}}}\left(a|\gamma ,X,I\right)=\frac{1}{\sqrt{2\pi }\sigma a}\exp\left\{-\frac{1}{2\sigma^{2}}\log^{2}\left[a/\gamma d_{ij}\left(X\right)\right]\right\}\;.$$ (12)

In both error models, *σ* quantifies the error of the Hi-C distance measurements.

#### Modeling single-cell Hi-C contacts with a logistic function

As an alternative to distance-based modeling, approaches directly based on the contact information have been proposed [7, 17]. Whenever two beads come closer than a distance threshold *d*_c_, the corresponding loci are in contact; we set *d*_c_ = 1.5 × *a*. Because we employ gradient-based methods in our sampling approach, we need to transform the binary contact constraints into a smooth function of the distance. We use a logistic function with steepness *α* to measure to which extent a contact is formed:

$$\mathrm{Pr}\left(D|X,I\right)=\prod_{(i,j)\in C}\frac{1}{1+\exp\{\alpha \left(d_{ij}\left(X\right)-d_{c}\right)\}}.$$ (13)

For large *α*, the logistic function approaches a step function. We set *α* = 200/*a* in our simulations.

Our model is reminiscent of logistic regression, a technique to fit linear models to binary data arising in classification problems. Here, the two classes correspond to contacts and non-contacts between two loci. However, in contrast to standard applications of logistic regression, only the first of the two classes is observed, because single-cell Hi-C gives us only information about contacting loci; the absence of a contact cannot be interpreted as the observation of a non-contact. With this degenerate data set, the regression parameters themselves remain undefined. Therefore, we have to fix the regression parameters *α* and *d*_c_. One possibility to estimate these parameters would be to combine single-cell with ensemble Hi-C data: the absence of ensemble Hi-C counts for pairs of mappable loci could, with some caution [41], serve as an observation of a non-contact. However, we have not explored this possibility further in the paper.

#### Modeling two-copy chromosome contacts using ambiguous distance restraints (ADRs)

When modeling the structure of diploid chromosomes, an observed single-cell Hi-C contact is ambiguous in the sense that it could be formed in either of the two copies. To deal with this ambiguity, we adopt an idea from NMR which is commonly used to work with distances that are ambiguous due to chemical shift degeneracy. Ambiguous distance restraints (ADR) compute an effective distance resulting form all alternative possibilities [35]. In the context of chromosome structure inference, we have two copies of the chromosome with conformations *X*_1_ and *X*_2_. The effective distance between beads *i* and *j* is computed as an *r*^−6^-average:

$${\bar{d}}_{ij}\left(X_{1},X_{2}\right)={\left[\;d_{ij}{\left(X_{1}\right)}^{-6}+d_{ij}{\left(X_{2}\right)}^{-6}\;\right]}^{-\frac{1}{6}}$$ (14)

where *d*_*ij*_(*X*_*k*_) is the distance between loci *i* and *j* in the *k*-th copy of the chromosome. The effective distance Eq (14) is fed into the logistic model (13) for Hi-C contacts such that the resulting likelihood function for diploid chromosomes is

$$\mathrm{Pr}\left(D|X_{1},X_{2},I\right)=\prod_{(i,j)\in C}\frac{1}{1+\exp\{\alpha \left({\bar{d}}_{ij}\left(X_{1},X_{2}\right)-d_{c}\right)\}}\;.$$ (15)

This model implements a soft version of a logical *OR*: If both *d*_*ij*_(*X*_1_) and *d*_*ij*_(*X*_2_) are smaller than the cutoff *d*_c_, the effective distance ${\bar{d}}_{ij}$ is also smaller than the contact distance. If the contact is formed in only one of both structures, the contribution from the large distance is negligible such that the effective distance roughly corresponds to the inter-bead distance in the structure that forms the contact. If none of the structures forms the contact, the effective distance will be larger than *d*_c_ and the contact will be violated.

For single-cell Hi-C data of diploid chromosomes it makes sense to use an *OR* rather than an *XOR* operation to explain the contacts. This is again a consequence of the fact that the absence of a single-cell Hi-C contact is not necessarily indicative of the loci being further apart than the contact distance *d*_c_. This is demonstrated by structures of the X chromosome calculated with contacts from six different cells. S3 Table shows that even though the structure ensembles differ in many aspects (see S4 Fig) there is a fraction of contacts (∼30%) that is formed across different cells. The possibility that a contact might be formed in only one of the two copies is captured by both *OR* and *XOR*. However, it could be that a contact which is observed for one of the copies is also compatible with the structure of the other copy, but not observed due to the limited coverage of single-cell experiments. An *XOR* operation would not allow for this possibility.

*Test calculation with incomplete distance information.* To get a better understanding of the strengths and shortcomings of our approach to model two-copy chromosomes, we ran test calculations on small protein structures that have the same size, but different topology. We used ubiquitin (PDB code 1ubq) and an NMR structure of the HRDC domain (PDB code 2ma1) as test systems. Ubiquitin is 76 amino acids in length, the HRDC domain is 75 amino acids long. By ignoring the last amino acid of ubiquitin, both structures can be represented by 75 beads located at the C*α* positions.

As a first test, we ran calculations with distance data. For both protein structures, we used all distances smaller than 10Å as input resulting in 547 distances for ubiquitin and 504 distances for the HRDC domain. ISD calculations for the invidual proteins showed that this incomplete distance information is sufficient to obtain a highly accuracte structure model. For ubiquitin, the mean structure achieved an RMSD of 0.6 Å to the correct structure. For the HRDC domain, we obtained a model of even higher accuracy with an RMSD of 0.4 Å.

Next we combined both sets of distances and tried to reconstruct the structure of ubiquitin and the HRDC domain simultaneously by the use of ambiguous distance restraints. Indeed, ISD was able to disentangle the distance and to obtain fairly accurate models of ubiquitin and the HRDC domain. S13A–S13D Fig show the ISD models in comparison with the correct structures. For ubiquitin, we achieved an overall RMSD of 2.1 Å. For the HRDC domain, the RMSD is 2.5 Å. The accuracy of the models is compromised by mixing the distance data, but it is clearly possible to reconstruct models of moderate accuracy by the use of ADRs.

*Test calculation with sparse contact data.* Next, we investigated whether also sparse contact information is sufficient to reconstruct two structural models from mixed experimental contacts. We computed contact data by keeping those pairs of C*α* positions whose distance is smaller than 4 times the van der Waals radius, which resulted in 323 contacts for ubiquitin and 313 contacts for the HRDC domain.

First, we ran ISD on the individual contact data. In both cases, we obtained ensembles that were close to the correct structure, but which also contained mirror images. The RMSD of the average structures is 2.2 Å (ubiquitin) and 1.9 Å (HRDC domain). To test if the use of ADRs is capable of disentangling mixed contact data, we combined the contacts from ubiquitin and the HRDC domain into a single set of 636 contacts, which we modeled in the same way as the diploid chromosome data. S11E–S11H Fig shows the average structures that we obtained by using the ambiguous contact model. Although the accuracy of the models is highly reduced (RMSD: ubiquitin 5.2 Å, HRDC domain 4.5 Å) the topology of the models is still correct. This illustrates that in principle contact data for diploid chromosomes can be separated into two sets of contacts that encode the single chromosome structures. When increasing sparsity of the contact data, this demixing becomes more and more challenging.

### Posterior sampling

Because the full posterior probability Pr(*X*, *θ*|*D*, *I*) (or Pr(*X*_1_, *X*_2_, *θ*|*D*, *I*) in case of diploidy) is of a non-standard form, we have to use Markov chain Monte Carlo (MCMC) sampling to draw representative chromosome conformations and model parameters that we can then use to compute average values and error bars. Gibbs sampling [44] is an iterative MCMC algorithm that draws from the posterior probability by cycling over successive steps that alternate between sampling of the conformational degrees of freedom and the model parameters:

$$\begin{array}{cc} X^{\left(t+1\right)}∼ & \mathrm{Pr}\left(X|\theta^{\left(t\right)},D,I\right)\propto \mathrm{Pr}\left(D|X,\theta^{\left(t\right)},I\right)\times \mathrm{Pr}\left(X|I\right)\\ \theta^{\left(t+1\right)}∼ & \mathrm{Pr}\left(\theta |X^{\left(t+1\right)},D,I\right)\propto \mathrm{Pr}\left(D|X^{\left(t+t\right)},\theta ,I\right)\times \mathrm{Pr}\left(\theta |I\right) \end{array}$$ (16)

This permits the use of adequate samplers for each model parameter.

#### Sampling of chromosome structures

An essential step in simulating Pr(*X*, *θ*|*D*, *I*) is to draw the conformational degrees of freedom *X* for fixed model parameters *θ*. This is challenging because we need to generate a random sample from a 3*N* dimensional distribution over all bead positions *x*_*n*_, which influence each other in a non-trivial way.

If there are multiple structures that are consistent with the distance restraints and the prior information, the conditional posterior distribution Pr(*X*|*θ*, *D*, *I*) will exhibit multiple peaks. Furthermore, the bead positions are highly correlated, because a displacement of one bead will exert forces not only on its direct neighbors along the fiber, but on all spatially close-by particles because of the volume exclusion term and the Hi-C contact restraints.

A large class of methods for sampling from general probability distributions are Markov Chain Monte Carlo (MCMC) algorithms, which construct a Markov chain whose unique limiting distribution is the distribution one aims to simulate. Simple MCMC methods such as the Metropolis-Hastings algorithm [45, 46] produce highly correlated samples. We therefore use Hamiltonian Monte Carlo (HMC, [29]), which uses the gradient of the log-probability to produce efficient proposals with high acceptance probability. HMC runs a short molecular dynamics (MD) simulation to efficiently produce proposal conformations of the chromosome.

Three parameters have to be set when using HMC for sampling. First, since HMC introduces fictitious momenta for each conformational degree of freedom, a mass matrix has to be specified. Second, the equations of motion solved during the short MD simulation are usually not analytically integrable and have to be solved by numerical integration schemes that require a discretization timestep. Third, the length of the MD trajectory needs to be chosen.

Rules-of-thumb to set these parameters exist [47], but require previous knowledge about the sampled probability distribution. We thus resort to heuristics. We set the mass matrix to unity, the trajectory length to 250 MD steps and determine an integration timestep such that an average acceptance rate of 50% is achieved.

#### Sampling of model parameters

*Sampling the distance scaling factor.* If we model the Hi-C contacts as distances, we need to estimate an unknown scaling factor γ that relates the experimental distance to the bead diameter: *δ* = *a*/γ. Because the distance scale is a positive quantity, we use a Jeffreys’ prior for γ (i.e. the prior over the distance scale is uniform in logγ). When using the Gaussian error model with a flat plateau, the one-dimensional sampling distribution for γ is of a non-standard form; we employ the Metropolis-Hastings algorithm to sample this parameter from its posterior distribution. In the case of the lognormal error model, the sampling distribution for γ is a lognormal distribution, from which sampling can be performed by using built-in random number generators.

*Sampling of error parameters.* How error parameters are best sampled again depends on the error model. For the lognormal error model, the conditional distribution of the data weight *w* = *σ*^−2^ is a Gamma distribution for which sampling routines are readily available in many programming languages. In case of the Gaussian error model with a flat plateau, we use the Metropolis-Hastings algorithm to generate samples of *σ*.

#### Replica exchange Monte Carlo

Because the Gibbs sampler (Eq 16) can get trapped in a peak of the posterior distribution, we embed the Gibbs sampler in a replica exchange (RE) algorithm [30, 31]. RE introduces a fictitious temperature λ^−1^ that facilitates posterior sampling. A family of tempered posteriors is introduced:

$$p_{\lambda }\left(X,\theta \right)\propto {\left[\mathrm{Pr}\left(D|X,\theta ,I\right)\right]}^{\lambda }\mathrm{Pr}\left(X|I\right)\mathrm{Pr}\left(\theta |I\right)\;.$$ (17)

We let the inverse temperature λ vary from 0 (prior) to 1 (posterior). The λ-schedule is designed such that with increasing replica index, the likelihood is more and more downweighted, that is, in the “high-temperature” replicas, there is only a weak influence of the data and the prior distributions dominate. This prevents the simulation from getting trapped in high-probability regions, but typically requires parallel computing resources.

### Model comparison and estimation of marginal likelihoods

The normalizing constant Pr(*D*|*I*) in Eq 2 is the marginal likelihood or *model evidence*. It is generally hard to calculate Pr(*D*|*I*), which is a high-dimensional integral over all unknown parameters *X* and *θ*. A great advantage of MCMC methods is that they can sample from *unnormalized* probability distributions. Therefore, the evidence Pr(*D*|*I*) does not need to be known when applying MCMC to sample from the ISD posterior. Although knowledge of Pr(*D*|*I*) is not necessary for parameter estimation (i.e. sampling of *X* and *θ*), the model evidence is crucial, if we want to do *model comparison*, i.e. if we want to quantify, for example, if the chromatin beads should exhibit only repulsive forces or both repulsive and attractive forces. The model evidence Pr(*D*|*I*) reflects how well the chosen likelihood and prior distributions describe the Hi-C data *D* and allows us to rank different modeling assumptions in the light of the data. To calculate the evidence, we use histogram reweighting techniques [48–50], which allow to use all samples from a RE simulation to estimate the model evidence in replica with high accuracy.

We used similar techniques to study the properties of the chromosome model (see Chromosome models). Because the prior alone prefers extended chromosome conformations, we imposed an additional radius of gyration term whose strength is varied smoothly via the inverse temperature λ of the replicas:

$$p\left(X|\lambda \right)\propto \exp\{-\lambda R_{g}\left(X\right)-k_{\text{bb}}E_{\text{bb}}\left(X\right)-k_{\text{nb}}E_{\text{nb}}\left(X\right)\}\;.$$ (18)

This RE simulation allowed us to explore all degrees of compactness of the chromatin fiber by varying λ between λ = 0 and λ = 500 using 64 replicas. The same histogram reweighting techniques that we used to calculate model evidences allowed us to obtain a precise estimate of the entropy as a function of *R*_g_. The entropy measures the number of conformations with a certain radius of gyration and is shown in Fig 1A.

### Analysis of structure ensembles

Structure ensembles reconstructed from single-cell Hi-C contacts are typically less well defined than NMR structure ensembles. Therefore traditional measures to characterize and compare structure ensembles such as RMSDs are of limited use.

#### Comparison of distance matrices

The structural properties of an ensemble of chromosome conformations can be assessed by analyzing the distances matrices computed for all members of the ensemble. This does not require a structural superposition of the ensemble members and also works in the presence of mirror images. We mainly use the sample mean and standard deviation over all distances matrices from one ensemble for comparison with another ensemble. More formally, let a posterior ensemble contain *M* chromosome structures $X_{m}=\left(x_{1}^{m},\ldots ,x_{n}^{m}\right)$ with *m* = 1, …, *M*. We first calculate the distance matrix *D*_*m*_ for every ensemble member. The elements of *D*_*m*_ are:

$${\left(D_{m}\right)}_{ij}=∥x_{i}^{m}-x_{j}^{m}∥\;.$$

We compute the entry-wise arithmetic means and standard deviations by

$$\bar{D}=\frac{1}{M}\sum_{m=1}^{M}D_{m}\;,\;\sigma \left(D\right)=\sqrt{\frac{1}{M}\sum_{m=1}^{M}{\left[D_{m}-\bar{D}\right]}^{2}}$$

where the squaring and the square-root are understood as element-wise operations.

For a pair of beads *i* and *j* that show an experimental contact, we expect that the entries of the average distance matrix $\bar{D}$ that are close to (*i*, *j*) are small compared to the average distance between pairs for which no contact was observed. The standard deviation *σ*(*D*) indicates how well the distance between all pairs of beads is determined by the data and the prior information. In regions without any data, for example beads close to the centromere, we expect a high standard deviation.

#### Diagonal permutation test on distance matrix correlations

The agreement of two structure ensembles can be assessed by comparing the corresponding average distances matrices. The cross-correlation coefficient of two distance matrices provides a measure of the similarity of the structure ensembles, but since the entries in the distance matrices are highly dependent, it is unclear if the correlation is statistically significant. The Mantel test [51, 52] aims to assess the statistical significance of the correlation between two distance matrices. The Mantel test relies on random permutations of rows (and corresponding columns) of one matrix to generate null models whose correlation with the other matrix is calculated. From the resulting set of correlations, a P-value is derived. The P-value indicates how likely it is to obtain the correlation coefficient between the original matrices by chance. However, recently Guillot *et al.* [53] showed that the Mantel test is only of limited use, because permutations of rows and columns destroy the autocorrelation within a matrix, which results in an underestimation of the P-value. Underwood *et al.* [54] proposed a diagonal permutation test which permutes matrix entries only within the diagonal they are part of. In our context, this procedure boils down to permuting entries only within the set of pairwise distances between beads with a genomic distance of at most *i* beads. Sequential correlations are preserved by this permutation procedure, which allows us to compute meaningful P-values. All reported correlations between average distance matrices are significant with at most *p* < 0.0001 (based on 10^4^ random shufflings of one of the distance matrices). S14 Fig confirms that the cross-correlation between two distance matrices is indeed an adequate measure of structural similarity.

#### Cluster analysis

Structural ensembles were clustered using spectral clustering [55]. For every pair of chromosome conformations in the ensemble we computed the standard RMSD (excluding the unmappable regions). The RMSD matrix was then transformed to a similarity matrix using an exponential transform, i.e.

$$s_{ij}=\exp\left\{-\beta R_{ij}\right\}$$

where *R*_*ij*_ is the RMSD between conformations *i* and *j*, *β* > 0 is a scaling factor which we set to *β* = 1/*a*. We run spectral clustering for various choices of the number *K* of clusters. The clustering that achieves the highest silhouette score [56] is then inspected further.

### Test calculations for a three-dimensional Hilbert curve

To validate our structure calculation method, we ran several test calculations for a three-dimensional Hilbert curve comprised of 512 beads. We assumed a cutoff distance of 1.5 × bead radius, which resulted in a maximum of 3696 contacts (∼2.8% of the full distance matrix, which has 512 × 511/2 = 130816 elements in total).

With the complete set of observable contacts, we obtained an ensemble that comprised two principal conformers which are mirror images of each other. The first cluster shows an average RMSD of 0.09 bead radii from the correct model and is populated by 48% of all sampled structures. The second cluster achieves the same RMSD to the mirror image of the ground truth and is populated by the remaining 52% of all structures. This shows that ISD is capable of drawing correct samples from the posterior distribution: Distance data alone cannot distinguish between a 3D structure and its mirror image. Therefore, we expect that the original structure of the Hilbert curve and its mirror image should be present in the ISD ensemble with identical probability.

We also computed structures with sparsified versions of the full set of contacts by randomly selecting a smaller number of contacts. In the sparsified data sets, the number of contacts ranged from 3326 to 578 contacts corresponding to 2.8% to 0.44% of all distances (the sparsity of the contact data in case of the ISD bead model of the X chromosome at 500 kb resolution is 0.68%). For all data sets, we obtained an ensemble of structures that comprised two major conformers: an approximate version of the input structure and its mirror image. The average structures of both clusters are shown in S11 Fig. Even with as few as 0.44% of all distances, ISD inferred structures that showed the correct domain architecture. The RMSD ranged from 0.13 to 1.46 bead radii for both the cluster approximating the ground truth and the cluster showing its mirror image (see S12A and S12B Fig). Also the spread and population of the clusters were almost identical (S12C and S12D Fig).

These tests show that (1) ISD is capable of generating statistically valid ensembles, which is indicated by the fact that we obtained a bimodal posterior ensemble, in which the peaks corresponding to the original structure and its mirror image have identical spread and population (see S12 Fig). (2) ISD generates structure ensembles that reflect the quality/completeness of the data, because the precision of the ensemble decreases with increasing sparsity of the contact data (see S12C and S12D Fig).

### Comparison with existing methods for chromosome structure inference

To better judge the performance of ISD, we also computed structural models with other methods for chromosome structure inference for the Hilbert curve data.

#### Shortest-path reconstruction in 3D (ShRec3D)

**Fig 8:**
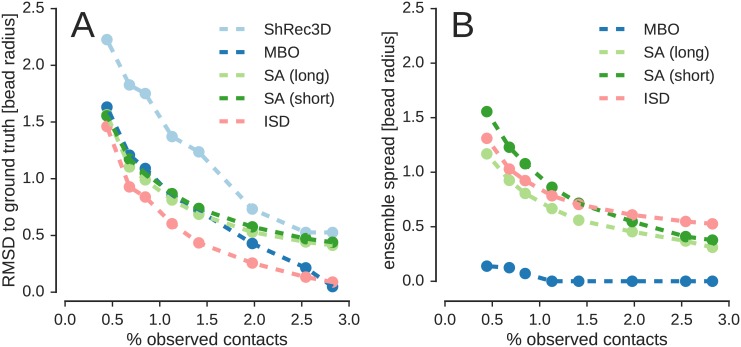
Comparison of ISD to existing methods for chromosome structure inference from single-cell Hi-C data. ShRec3D, MBO and the simulated annealing procedure (SA) by Nagano *et al.* (two versions of SA were tested: a long version based on the default annealing protocol and a short version with ten-fold faster annealing). **(A)**: Accuracy (RMSD to ground truth) achieved with all four methods. **(B)**: Precision (spread of structural ensemble) for MBO, SA and ISD (ShRec3D produces only a single model, therefore it is not possible to assess the precision of the ShRec3D model). Both accuracy and precision are quantified by RMSDs and increase when the RMSDs decrease.

We implemented the ShRec3D [19] algorithm in Python and applied it to the Hilbert curve data. With the complete set of observable contacts (3696 contacts, 2.8% of all distances) ShRec3D obtained a structure of reasonable accuracy with an RMSD of 0.53 bead radii to the ground truth. However, with increasing sparsity of the contact data the structure becomes increasingly inaccurate (see Fig 8A). At the sparsity level of the single-cell Hi-C data by Nagano *et al.* at 500 kb resolution (0.68% of all distances) the RMSD is 1.8 bead radii (for comparison, ISD achieves an RMSD of 0.9 bead radii).

An advantage of embedding algorithms such as ShRec3D is that the structural model is obtained by computing an eigendecomposition, which is very fast. However, the speed of the method comes at the prize of being quite restrictive in the prior assumptions that can be imposed on the chromosome fiber. Another serious limitation is that only a single structure is obtained (modulo reflections and rigid transformations). Therefore, it is not possible to make statements about the precision of the reconstructed chromosome structure.

#### Manifold-based optimization (MBO)

Manifold-based optimization (MBO) [20] can be viewed as a refined and more robust version of ShRec3D. MBO produces reconstructions of the Hilbert curve that are systematically more accurate than the models produced by ShRec3D (see Fig 8A). However, the structures found by ISD are still more accurate than the MBO models for most data sets. Only for the most complete data set (100% of all contacts, 2.8% of all distances), MBO produces a slightly more accurate model of the Hilbert curve with an RMSD of 0.05 bead radii, whereas the ISD structure achieves an RMSD of 0.09 bead radii. For all other data sets, ISD produces a more accurate model of the Hilbert curve.

In contrast to classical MDS, the optimization problem that MBO solves is no longer convex due to a rank constraint, which guarantees that the solutions live in 3D space. As a consequence, there are multiple minima which the optimizer locates. By running the optimization procedure multiple times from randomized initial structures, MBO produces a set of possible chromosome structures. However, the MBO ensemble does not have the same statistical foundation as the ISD ensemble. Because ISD uses a sampling algorithm to explore the posterior distribution, it produces a faithful representation of the information content of the data. This is reflected by a high correlation between the *precision* of the ensemble as measured by the average RMSD to the average structure and the *accuracy* (RMSD between ground truth and average structure), which reaches 99.7% in case of the ISD ensemble and drops to 86.3% for MBO.

Fig 8B also shows that the absolute value of the spread of the MBO ensemble totally underestimates the inaccuracy of the structure. For data sets that do not suffer too much from a lack of data (number of observed contacts > 1% of all distances), there is almost no variability in the MBO ensemble (the ensemble spread is ∼10^−4^ bead radii): MBO is over confident about the reliability of the chromosome structure. The reason is that at this moderate level of data sparsity the optimization problem is still well-defined, such that the optimizer always finds the same minimum structure (and its mirror image). As the completeness of the data dwindles, MBO starts to produce slightly more heterogeneous ensembles, but the ensemble spread is still more than an order of magnitude smaller than the accuracy. For the sparsest data set (0.44% of all distances), the spread of the MBO ensemble is still only 0.14 bead radii. Therefore, the spread of the MBO ensemble is not a meaningful indicator of the reliability of the reconstructed chromosome structure and seems to mostly reflect the difficulty of the optimization problem. The spread of the ISD ensemble, on the other hand, gives a reasonable estimate of the true accuracy of the ensemble.

#### Simulated annealing approach by Nagano *et al.* (SA)

We also applied the simulated annealing approach by [11] to the Hilbert curve data. We tested two versions of the SA approach: one using the default temperature schedule and a second simulation with a 10-fold faster annealing protocol. With sparse contact data, both versions of SA achieve a similar accuracy as MBO (see Fig 8A). With more complete data, SA performs similar to ShRec3D. For all data sets, both versions of SA are systematically less accurate than ISD. Similar to ISD and MBO, SA finds both the input Hilbert curve and the mirror image with equal probability.

Fig 8B shows that the precision of the SA ensembles is a good indicator of the reliability of the reconstructed structure. However, the exact spread of the SA ensemble depends on the length of the annealing schedule. With the 10-fold longer schedule, the SA ensembles are systematically tighter than with the short schedule. The reason is that SA is an optimization method that aims to locate the minimum of the objective function and not to adequately represent the uncertainty of the structure. With a longer annealing schedule, the chance of finding a structure that is close to the minimum increases, and thus the spread of the ensemble decreases. Therefore, the precision of SA ensemble does not only reflect the quality of the data, but is to some extent also determined by the computational effort that is spent on the minimization.

#### Computation times

One great advantage of embedding methods is that they are many orders of magnitude faster than a full ISD simulation. To give a rough estimate: A single HMC proposal generated by a trajectory of 250 MD steps takes ∼70% of the computation time of running ShRec3D. A typical ISD simulation consists of 50 replicas each of which executes many HMC sampling steps (10 × 250 MD steps between two replica transitions). We typically sample 1000 replica transitions, which amounts to a total number of 50 × 10 × 250 × 1000 = 1.25 × 10^8^ gradient evaluations / MD steps. On a single CPU this would take approximately 3.5 × 10^5^ the running time of ShRec3D. However, one has to keep in mind that running ShRec3D takes less than half a second at 500 kb resolution. Moreover, convergence of the replica simulation is usually very fast (in the order of 20 to 50 replica transitions), such that on a computer cluster a full ISD calculation can be run in one or two hours. If one is only interested in a single structure and does not aim to sample the posterior distribution exhaustively, structural models can be obtained by running only the HMC algorithm (without the overhead introduced by parallel tempering / replica exchange simulation), which takes only a few minutes and is of similar speed as the SA implementation by Nagano *et al*. A table listing the computation times for the different modes of running ISD can be found in S4 Table.

### Software

Simulations were performed using an extended version of the ISD library [57]. Analyses were carried out with Python scripts that rely only on widely used additional packages and the CSB toolbox [58]. Code and scripts for analysis are available at https://github.com/michaelhabeck/isdhic.

## Supporting Information

S1 Fig Comparison of distance matrices for different prior distributions. Pooled histogram of all distances involved in an experimentally observed contact **(A,D,G,J)**, average distance matrices **(B,E,H,K)** and standard deviations **(C,F,I,L)** for the contact model combined with various prior probabilities. **(A,B,C)** repulsive volume exclusion term; **(D,E,F)** repulsive term and FISH data; **(G,H,I)** Lennard-Jones potential; **(J,K,L)** Lennard-Jones potential and FISH data.
(PDF)

S1 Table Estimated model evidence. (PDF)

S2 Fig Pooled histogram of all distances involved in an experimentally observed contact. **(A)** contact model; **(B)** Gaussian with flat plateau; **(C)** lognormal model.
(PDF)

S3 Fig Relation between the weight of the lognormal restraint *w* = *σ*^−2^ and the estimated distance scale γ. (PDF)

S1 Appendix Full inference of distance-based models. (PDF)

S4 Fig Cluster analysis of the ISD ensemble. For details see caption of S5 Fig.
(PDF)

S5 Fig Cluster analysis of the ensemble calculated by [11]. **(A)** The left heatmap shows the raw RMSD matrix for all 200 ensemble members (blue: small pairwise RMSD, red: large pairwise RMSD). The right heatmap shows the same matrix with sorted rows and columns according to cluster membership where the clusters themselves are sorted by cluster size. Homogeneous blue squares along the diagonal indicate that the clusters are well defined and tighter than the off-diagonal rectangles whose color tends towards red, if the clustering is well-defined (high silhouette score). The ensemble is comprised of four principal clusters of approximately equal size (populations: 28, 26, 24, and 24%). The color bar indicates the RMSD in microns. **(B)** Silhouette score as a function of the number of clusters. **(C)** Members of the four clusters are shown in the top row (blue centromere to red telomere). The bottom row show tube representations of the cluster centers. The tube thickness indicates the positional uncertainty of the corresponding beads.
(PDF)

S2 Table Correlation between average distance matrices for six Th1 cells. Shown are the correlation coefficients between the average distance matrices (in %) for structure ensembles obtained from cell 1 to cell 6. Lower diagonal: ISD ensemble vs ISD ensemble. Upper diagonal: ISD ensemble vs ensemble by Nagano *et al.* Diagonal (shown in bold face): ISD ensemble vs ensemble by Nagano *et al.*
(PDF)

S3 Table Restraint violations in six Th1 cells. Percentage of restraints from different cells (columns) that are violated in structure ensembles (rows) with a tolerance of *a*/8.
(PDF)

S6 Fig Average distance matrices calculated from structure ensembles reconstructed from six Th1 cells. (PDF)

S7 Fig Average X-chromosome structures from structure ensembles reconstructed from six Th1 cells. (PNG)

S8 Fig Comparison of population Hi-C maps with contact frequencies derived from the ISD ensemble. Left: population Hi-C map from [11]. Right: Contact frequencies derived from the ISD ensemble based on single-cell data from cell 1.
(PDF)

S9 Fig Spatial distribution of epigenetic marks in the X chromosome. The cumulative number of *trans*-chromosomal contacts **(A)**, lamin B1 **(B)** interaction scores, H3K4me3 **(C)** and gene density **(D)** are calculated as a function of depth for the prior ensemble based on chain connectivity, volume exclusion and the radius of gyration term (blue histogram) and the posterior ensemble incorporating the contact restraints (red histogram). Lamin B1 scores, H3K4me3 and gene density were obtained from the Supplementary Data in Nagano et al. [11].
(PDF)

S10 Fig Average distance matrices calculated from structure ensembles reconstructed at 50 kb (A) and 500 kb resolution (B). (PDF)

S11 Fig Average structures of the 512-Hilbert curve reconstructed from incomplete contact information. Top two rows show the original structure **(A)** and conformers inferred from contact data of different levels of sparsity **(B-H)**. Corresponding mirror images are shown in the bottom rows **(I-P)**. In all cases, both conformers are almost equally populated. Number of contacts used in reconstruction 3696 **(B,J)**, 3326 **(C,K)**, 1848 **(D,L)**, 1478 **(E,M)**, 1108 **(F,N)**, 889 **(G,O)**, 578 **(H,P)**.
(PNG)

S12 Fig Accuracy and precision of the ISD ensemble as a function of the completeness of the input data. Tests were run on the 512-Hilbert curve with dwindling number of input data. Each ISD ensemble comprised two major clusters corresponding to the input curve and its mirror image. For each cluster, the accuracy was assessed by computing the RMSD to the input structure **(A)** / its mirror image **(B)**. The precision or ensemble spread is defined as the average RMSD to the cluster center and shown in panels **(C,D)**.
(PDF)

S13 Fig Disentangling mixed distance data from two protein structures. Ubiquitin is shown in the left panels, the HRDC domain is shown in the right panels. The top row shows the average bead models obtained from the mixed set of distances. The bottom row shows the average bead models obtained from mixed sets of sparse contacts. Ubiquitin models obtained with distance / sparse contact information are shown in panels **(A)** and **(E)**. The ground truth is shown in panels **(B)** and **(F)**. Models of the HRDC domain obtained with distance / sparse contact information are shown in panels **(C)** and **(G)**. The ground truth is shown in panels **(D)** and **(H)**.
(PDF)

S14 Fig Significance of distance correlations. Structure ensembles were calculated with a reduced set of contacts where the *n* longest range contacts have been ignored. The ISD ensembles based on the reduced set of data were compared with the ensemble obtained with the full data set to study the robustness of the ISD approach and the significance of the distance correlations. **(A)** Cross-correlation between average distance matrices as a function of the number of missing long-range contacts. **(B)** RMSD between the average structures of the ensemble obtained with all contacts and reduced number of contacts. **(C)** Correlation between correlation of distance matrices and RMSD.
(PDF)

S4 Table Computation times. Tests for the calculation of a single structure were done on a standard laptop with 2.90GHz Core i7 8-core processor with 16 GB memory. At a resolution of 500 kb, the test consisted of 1000 steps of HMC starting from an extended structure. At a resolution 50 kb, 10000 steps of HMC were run starting from an extended structure. To compute the posterior ensemble of the X chromosome, we ran the replica-exchange algorithm on a computer cluster using 50 CPUs. At a resolution of 500 kb, we simulated 200 replica transitions where each transition consisted of 10 steps of HMC each using 250 leapfrog integration steps. At a resolution of 50 kb, we ran 1000 replica transitions on 50 CPUs. It is possible to shortcut the computation of the high-resolution structure by starting from a low resolution model. The initial structure of the high-resolution model is obtained by using a 3D spline interpolation with 10-fold higher sampling. For the single structure calculation, it is sufficient to reduce the number of HMC steps to 1000. In case of the replica simulation, the correct ensemble is obtained after 200 replica transitions. The corresponding computation times are indicated by “500 kb + 50 kb” in the S4 Table.
(PDF)
