# Supplementary figures and images for: Inferential Structure Determination of Chromosomes from Single-Cell Hi-C Data
Source: PLoS Comput Biol. 2016 Dec 27;12(12):e1005292. doi: 10.1371/journal.pcbi.1005292 (PMC5226817; doi:10.1371/journal.pcbi.1005292)

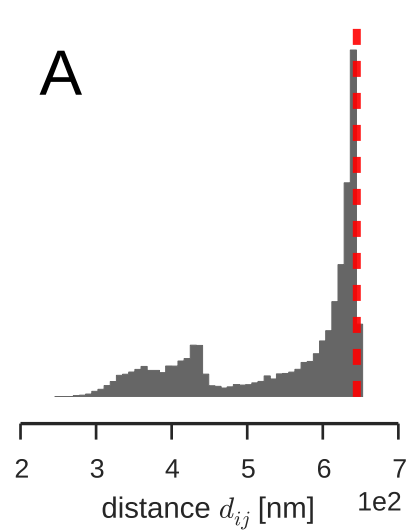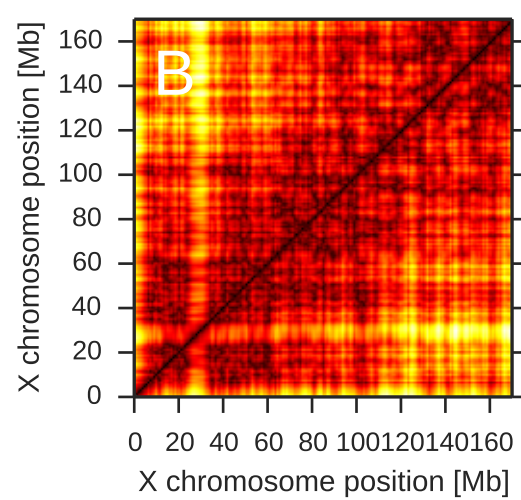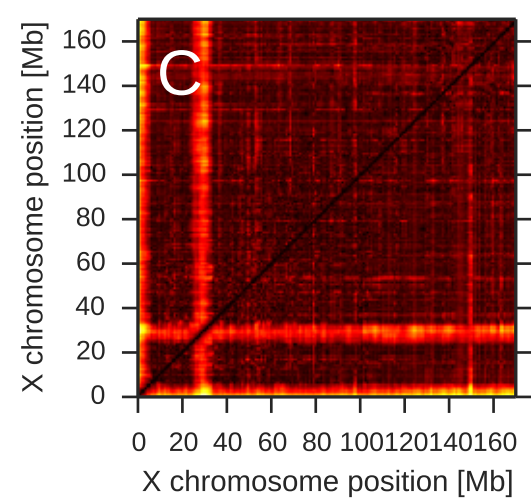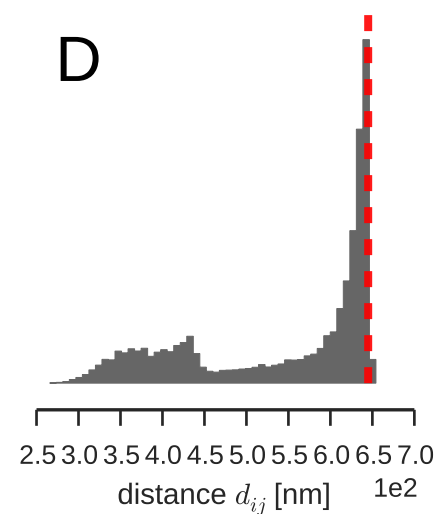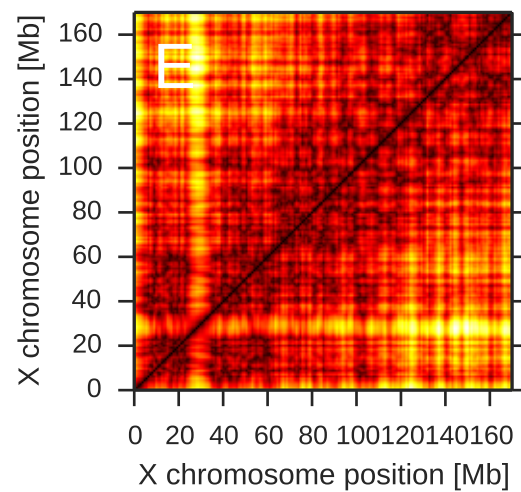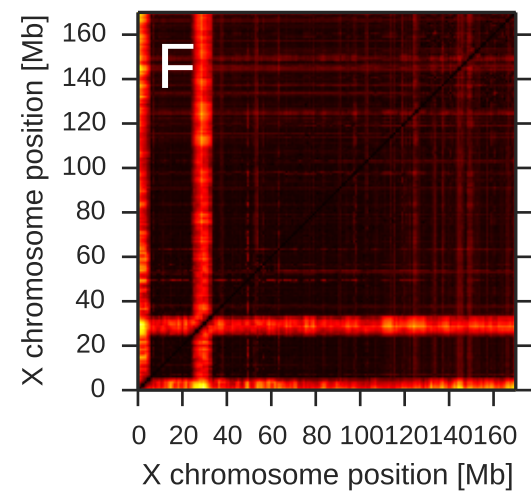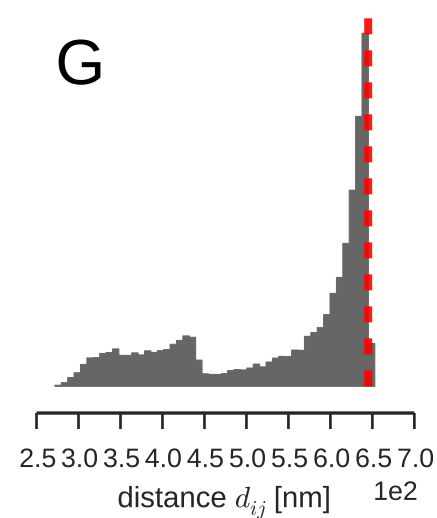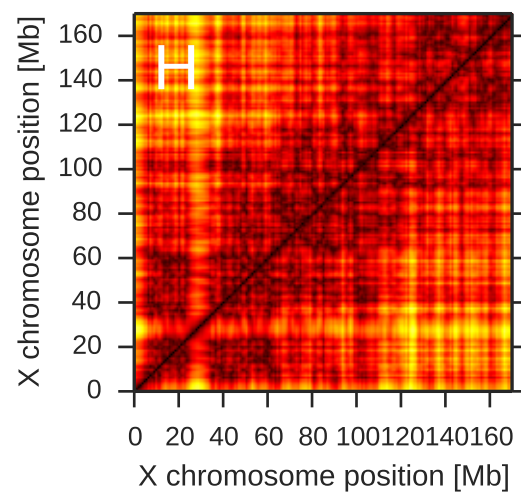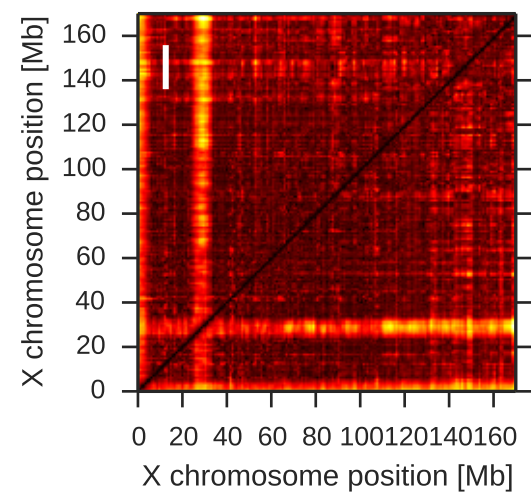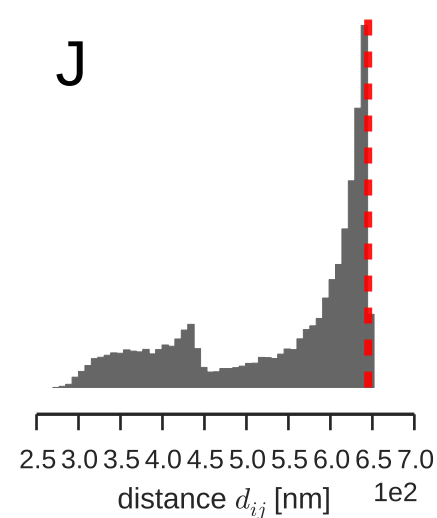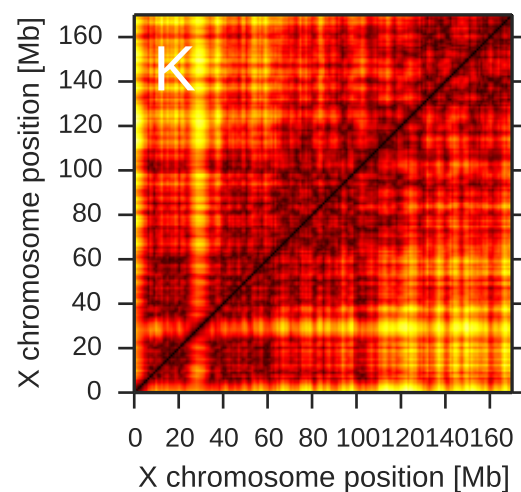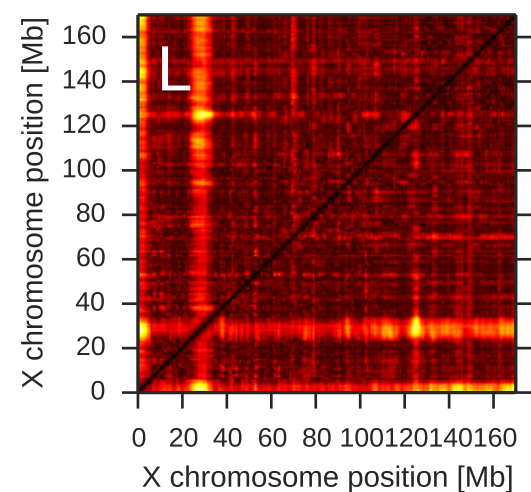

Supplement: S1 Fig — Pooled histogram of all distances involved in an experimentally observed contact (A,D,G,J), average distance matrices (B,E,H,K) and standard deviations (C,F,I,L) for the contact model combined with various prior probabilities. (A,B,C) repulsive volume exclusion term; (D,E,F) repulsive term and FISH data; (G,H,I) Lennard-Jones potential; (J,K,L) Lennard-Jones potential and FISH data. (PDF) [file pcbi.1005292.s001.pdf]

A

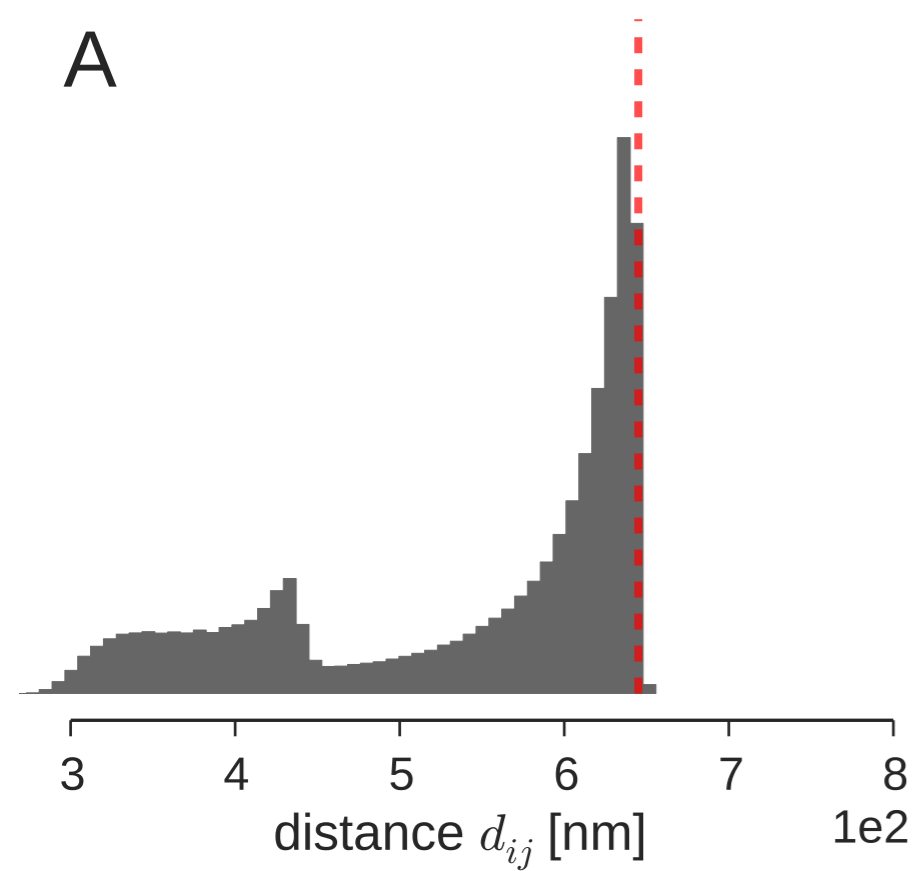

B

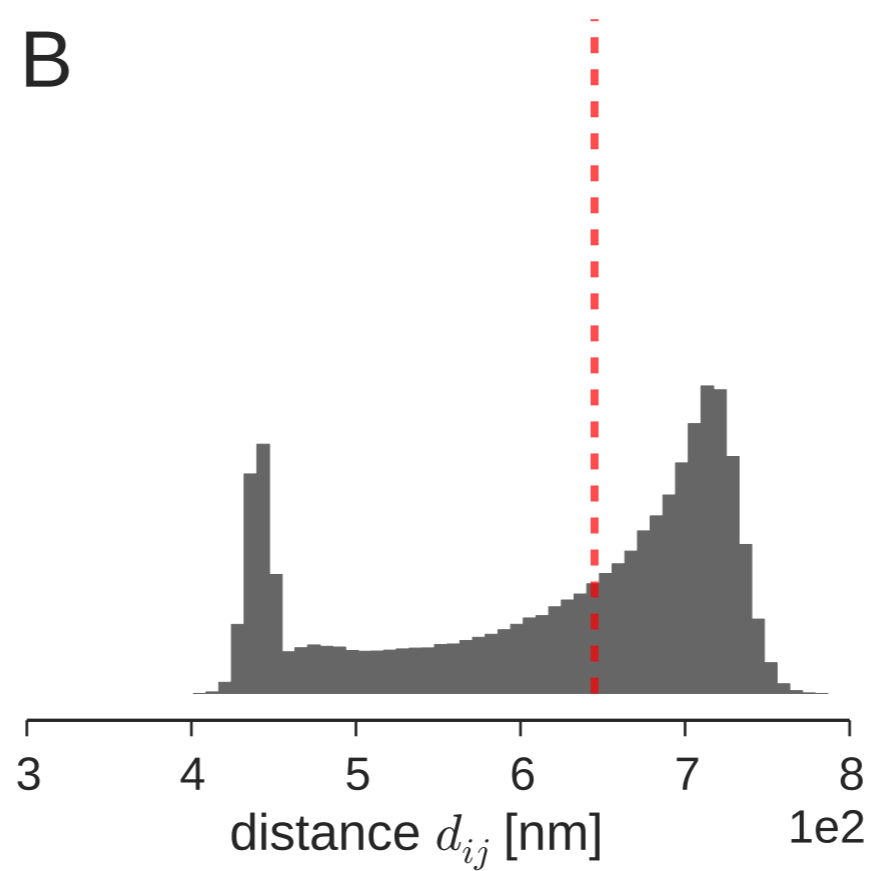

C

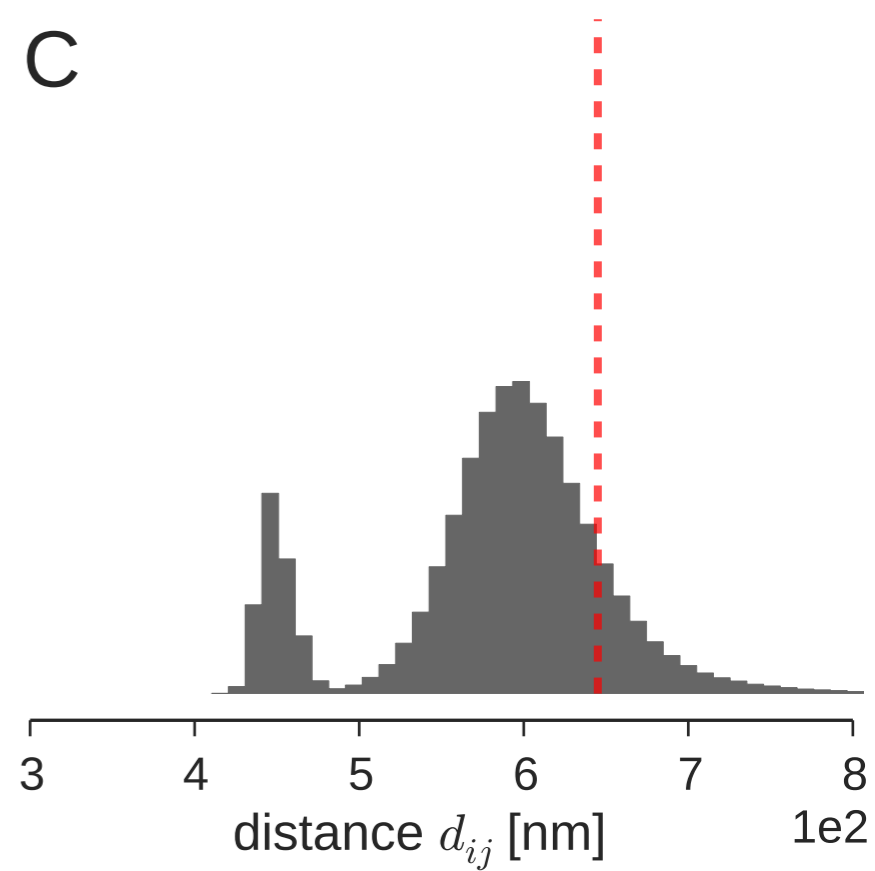

Supplement: S2 Fig — (A) contact model; (B) Gaussian with flat plateau; (C) lognormal model. (PDF) [file pcbi.1005292.s003.pdf]

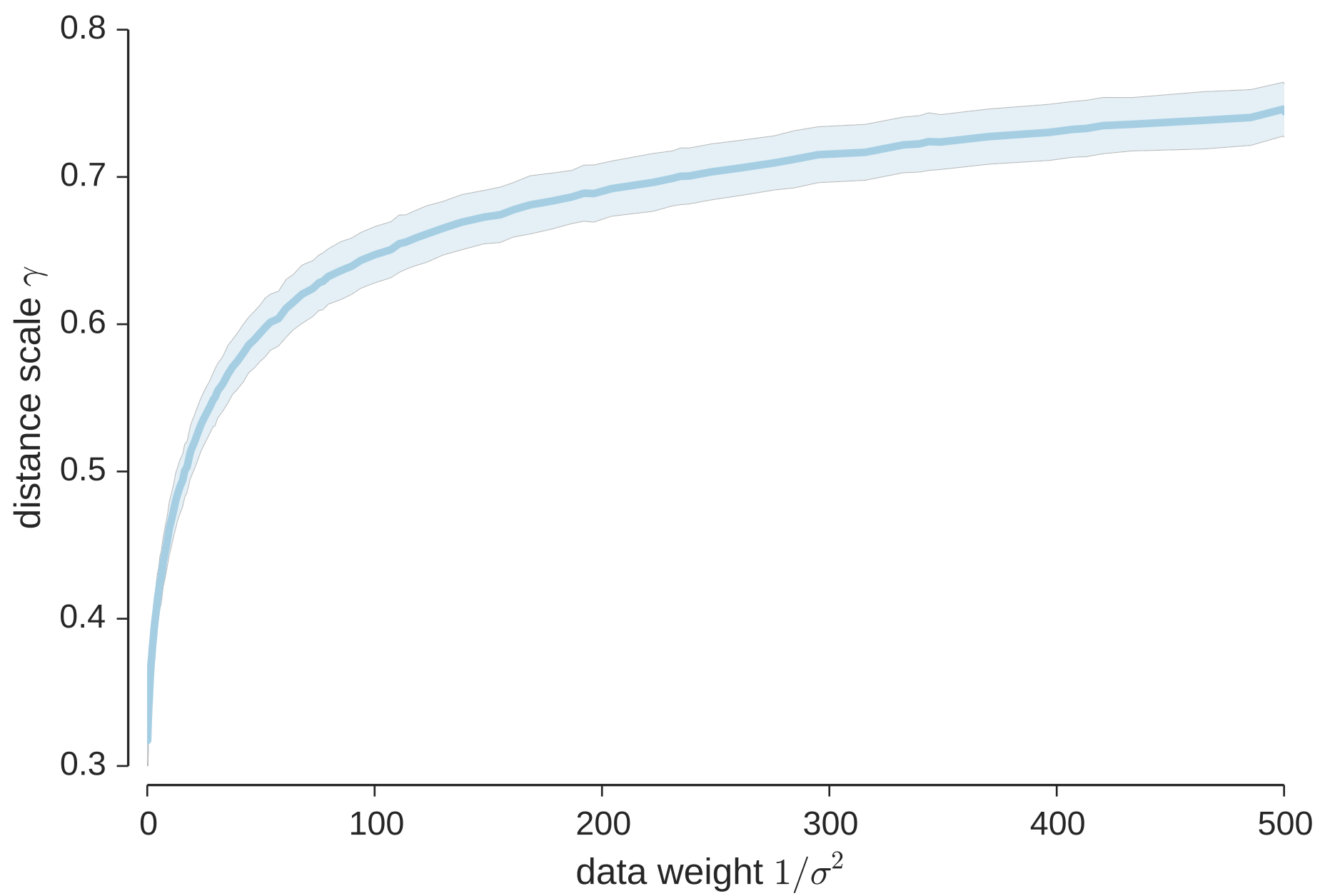

Supplement: S3 Fig — (PDF) [file pcbi.1005292.s004.pdf]

A

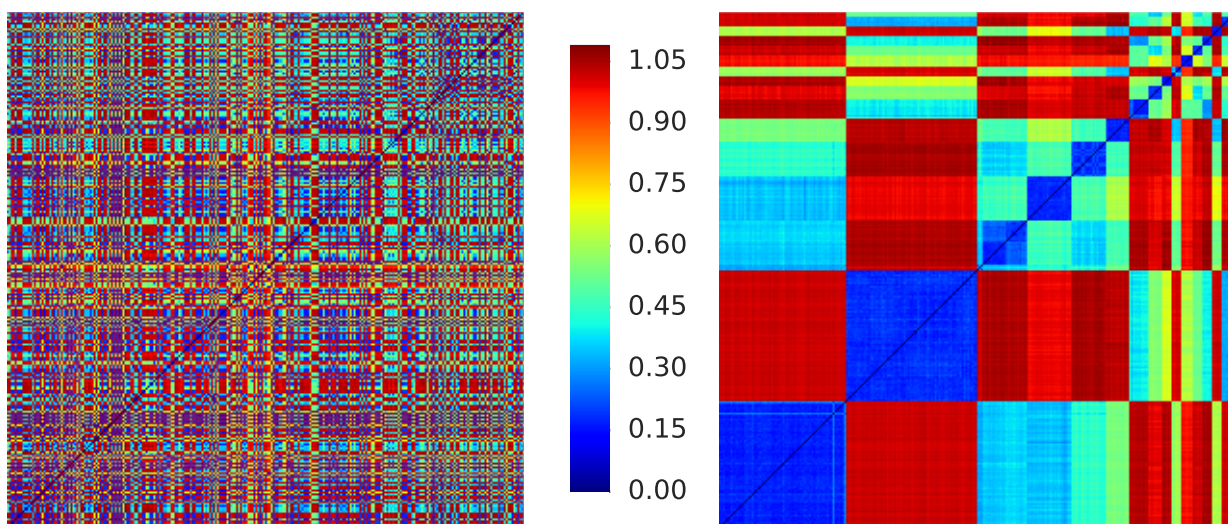

B

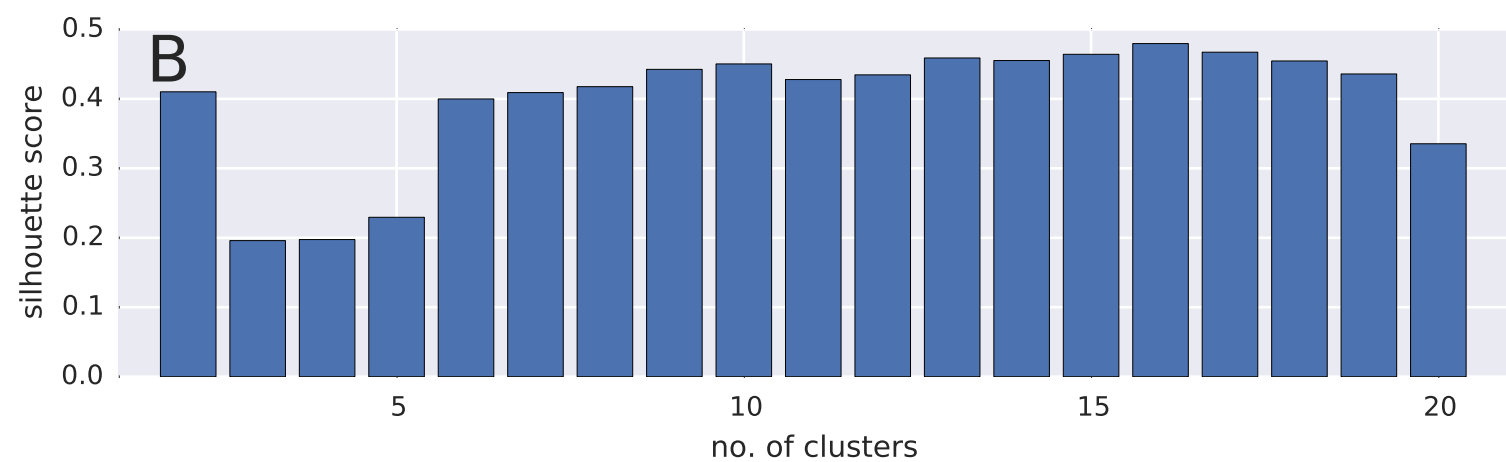

C

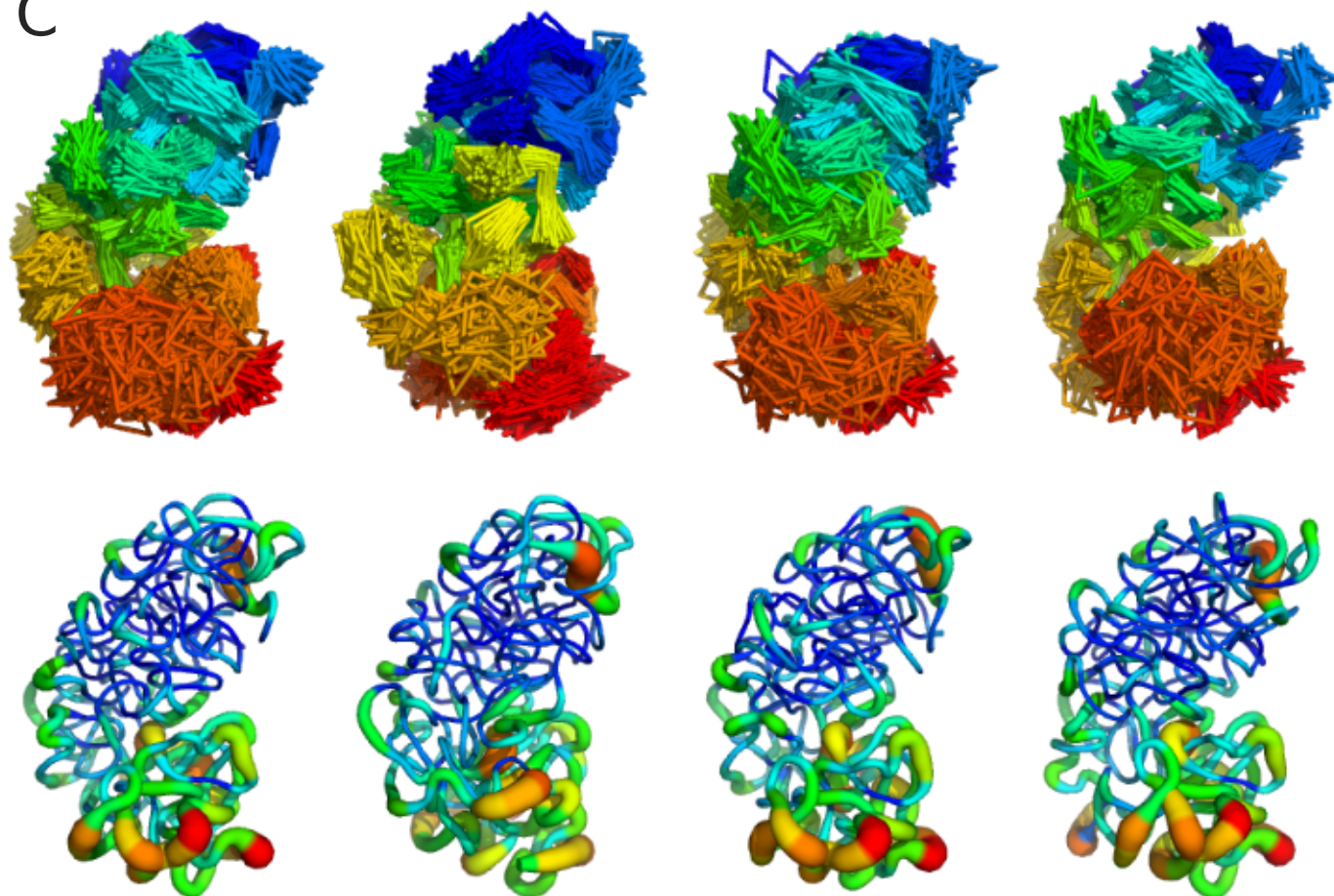

Supplement: S4 Fig — For details see caption of S5 Fig. (PDF) [file pcbi.1005292.s006.pdf]

A

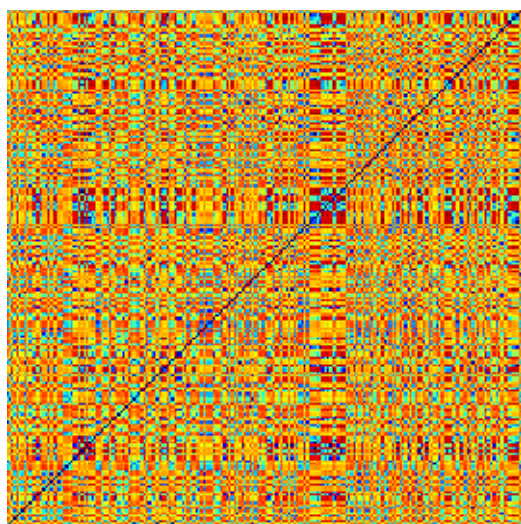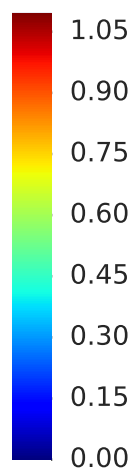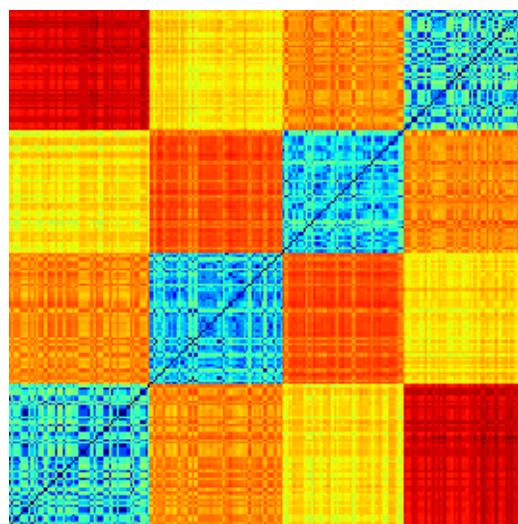

B

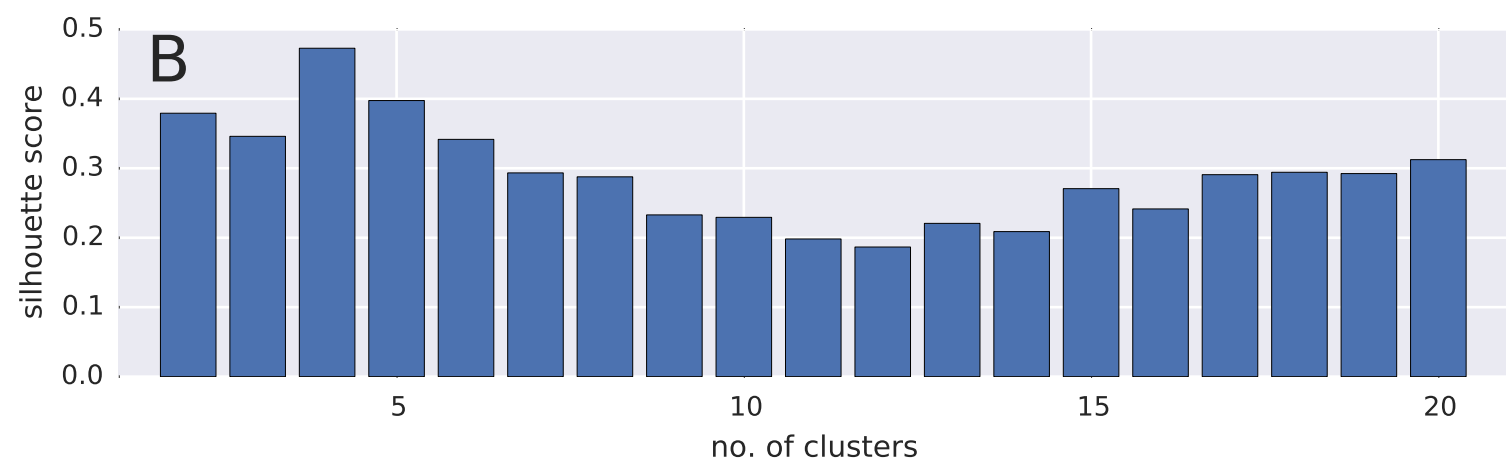

C

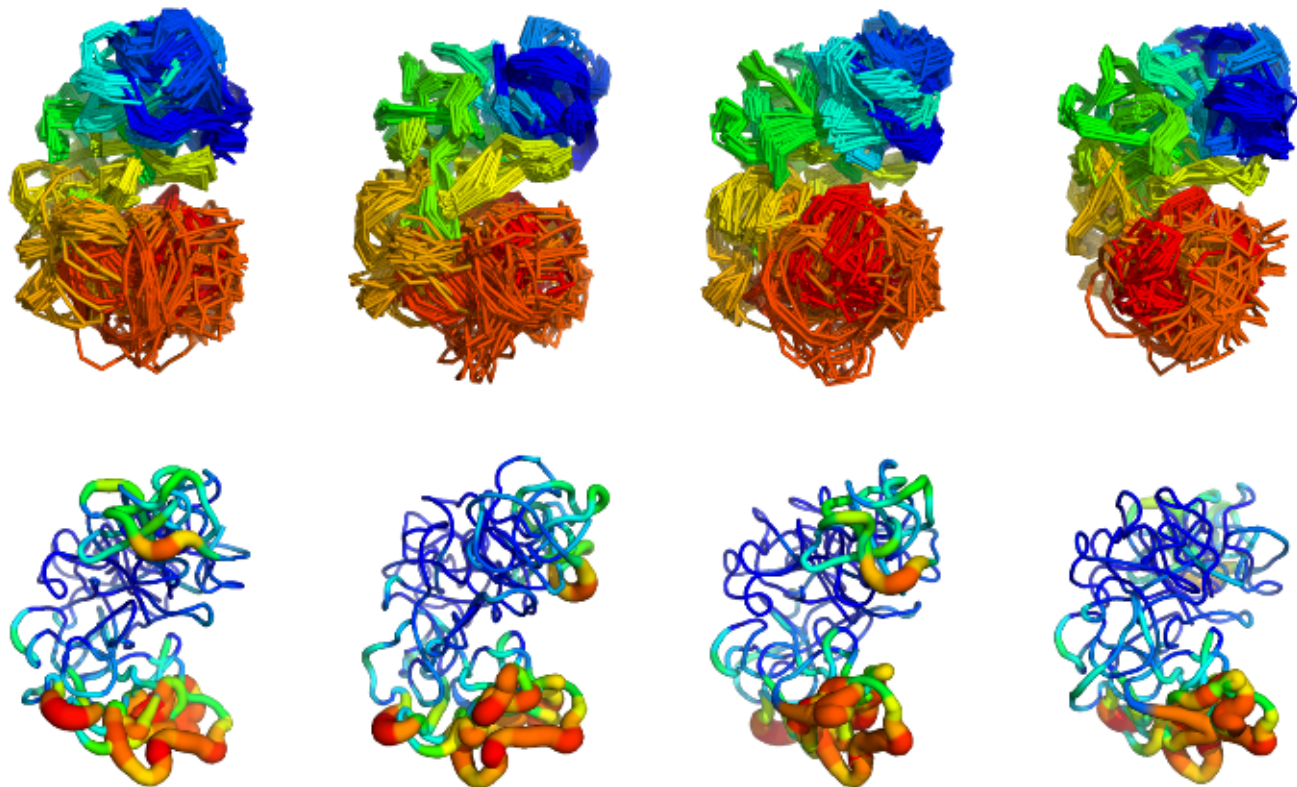

Supplement: S5 Fig — (A) The left heatmap shows the raw RMSD matrix for all 200 ensemble members (blue: small pairwise RMSD, red: large pairwise RMSD). The right heatmap shows the same matrix with sorted rows and columns according to cluster membership where the clusters themselves are sorted by cluster size. Homogeneous blue squares along the diagonal indicate that the clusters are well defined and tighter than the off-diagonal rectangles whose color tends towards red, if the clustering is well-defined (high silhouette score). The ensemble is comprised of four principal clusters of approximately equal size (populations: 28, 26, 24, and 24%). The color bar indicates the RMSD in microns. (B) Silhouette score as a function of the number of clusters. (C) Members of the four clusters are shown in the top row (blue centromere to red telomere). The bottom row show tube representations of the cluster centers. The tube thickness indicates the positional uncertainty of the corresponding beads. (PDF) [file pcbi.1005292.s007.pdf]

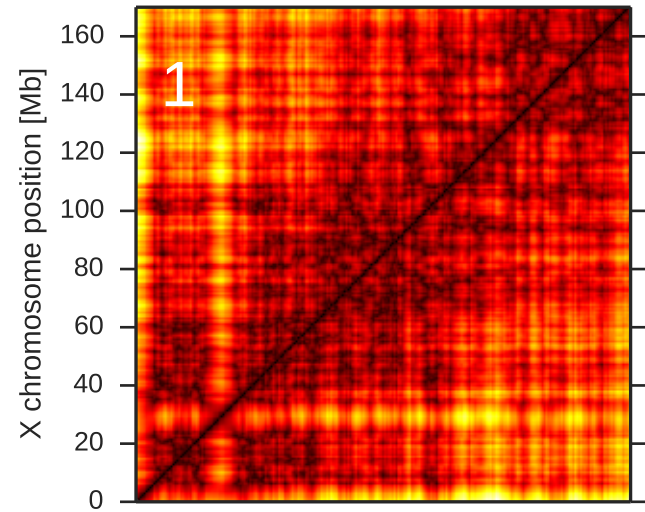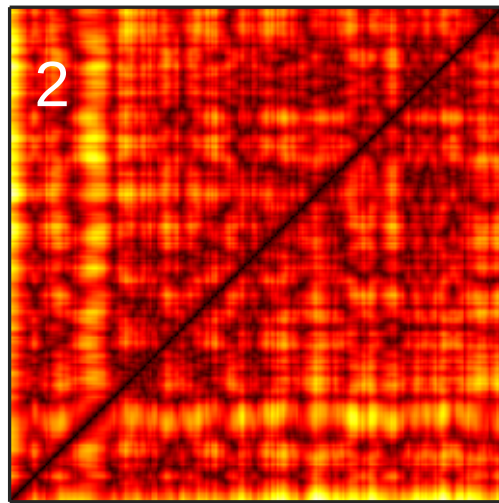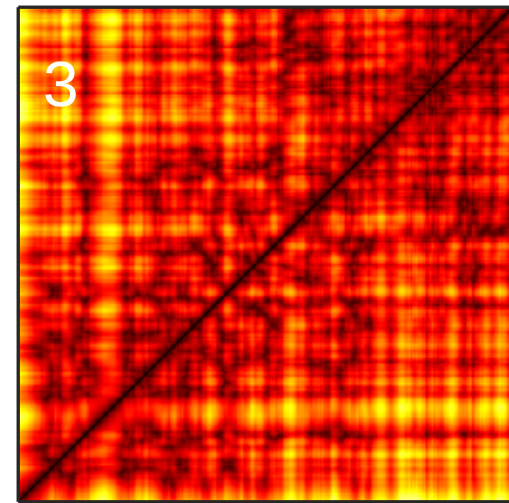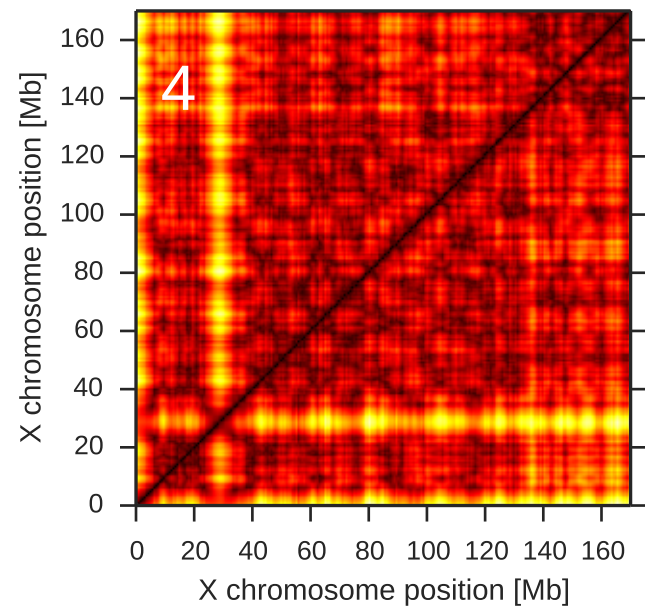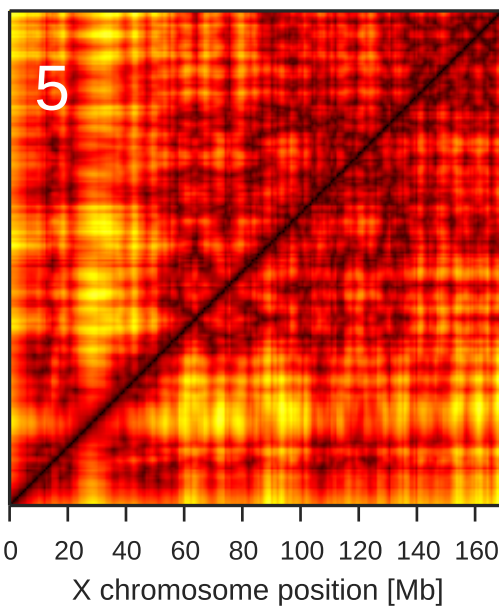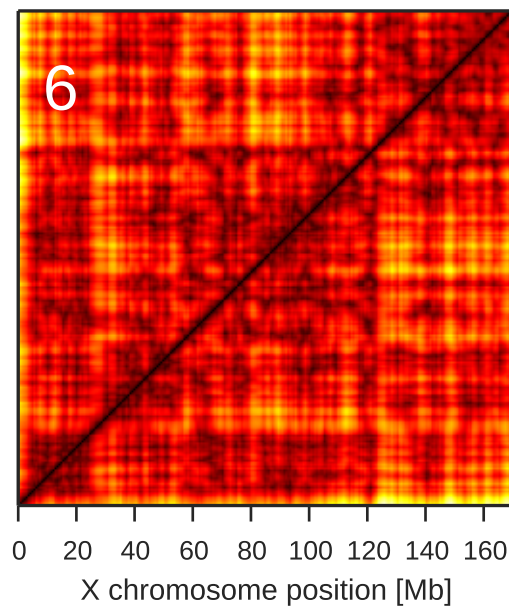

Supplement: S6 Fig — (PDF) [file pcbi.1005292.s010.pdf]

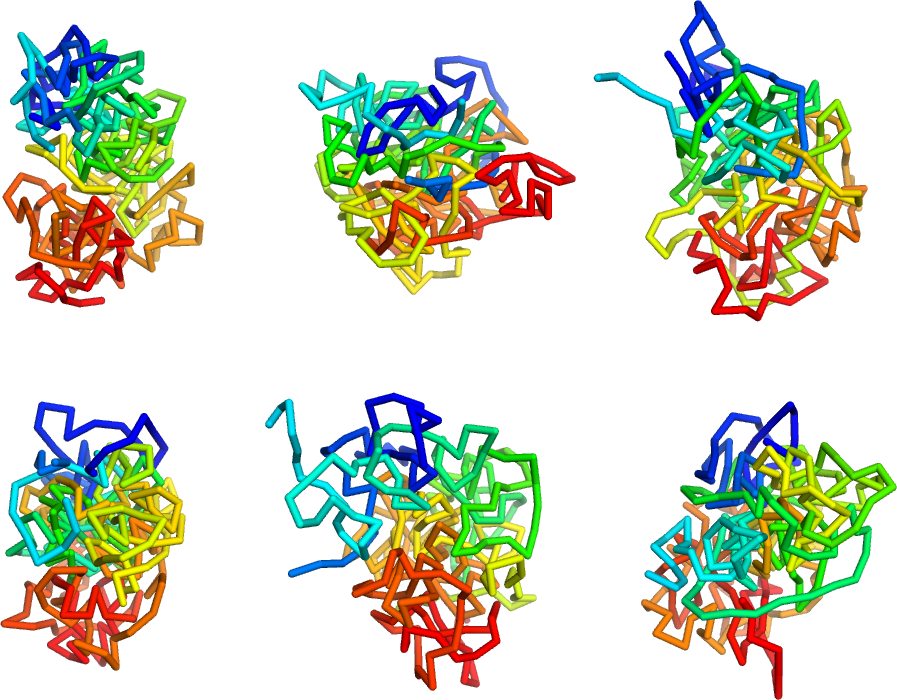

Supplement: S7 Fig — (PNG) [file pcbi.1005292.s011.png]

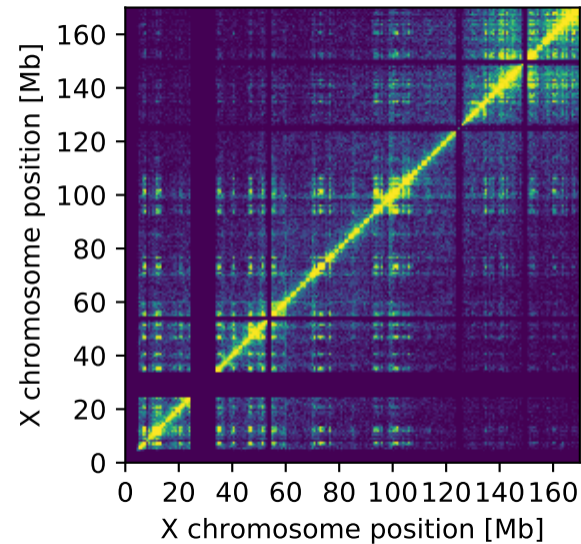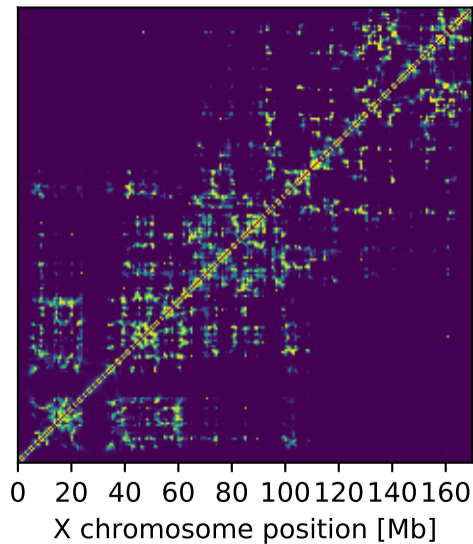

Supplement: S8 Fig — Left: population Hi-C map from [11]. Right: Contact frequencies derived from the ISD ensemble based on single-cell data from cell 1. (PDF) [file pcbi.1005292.s012.pdf]

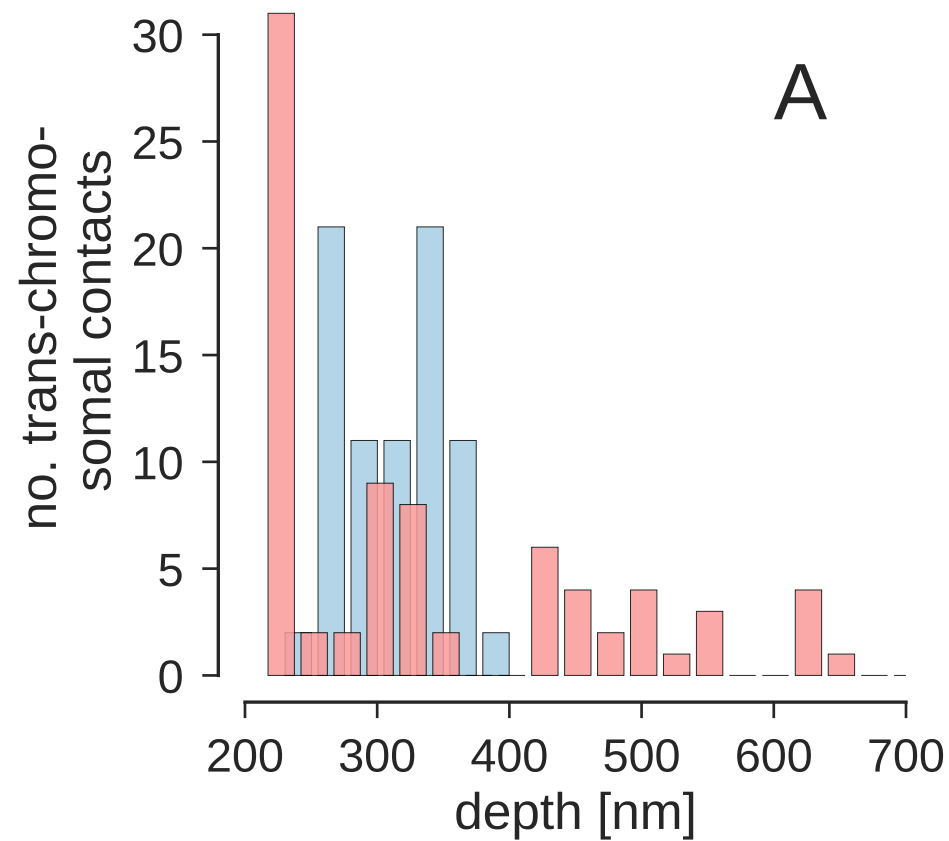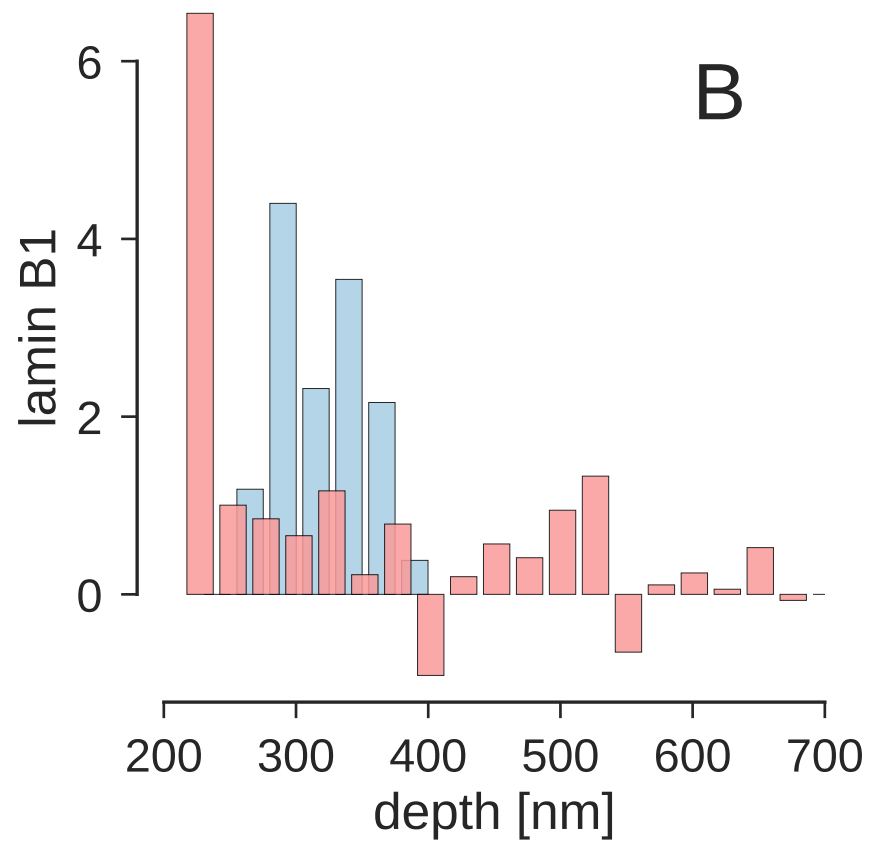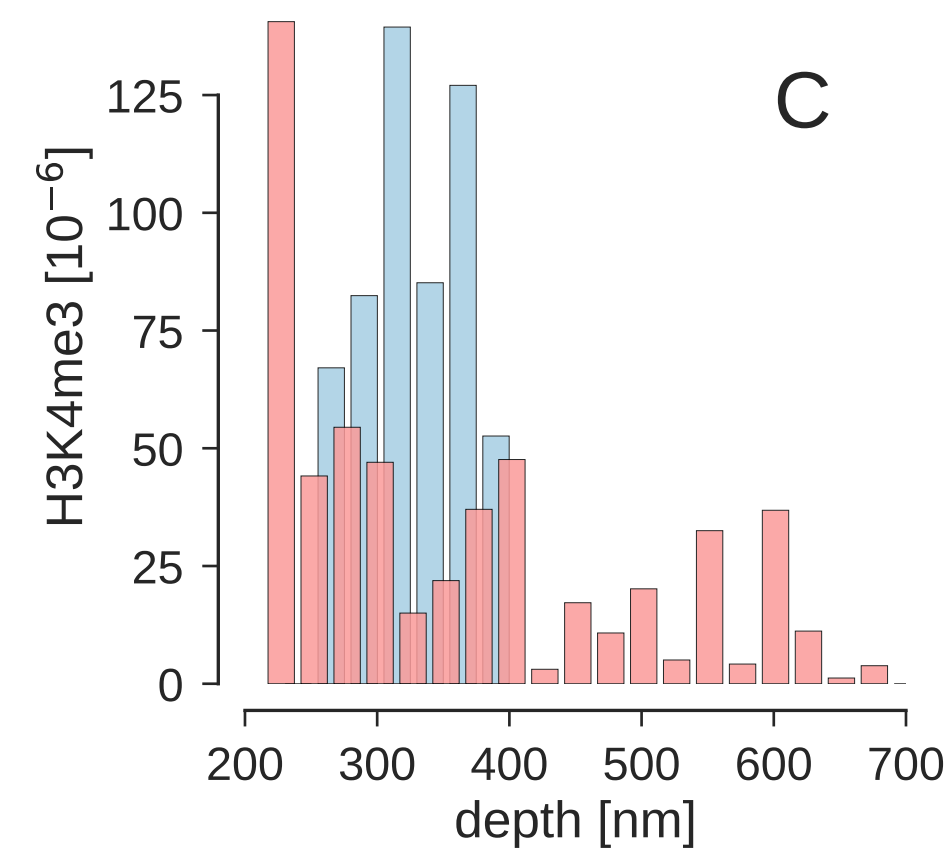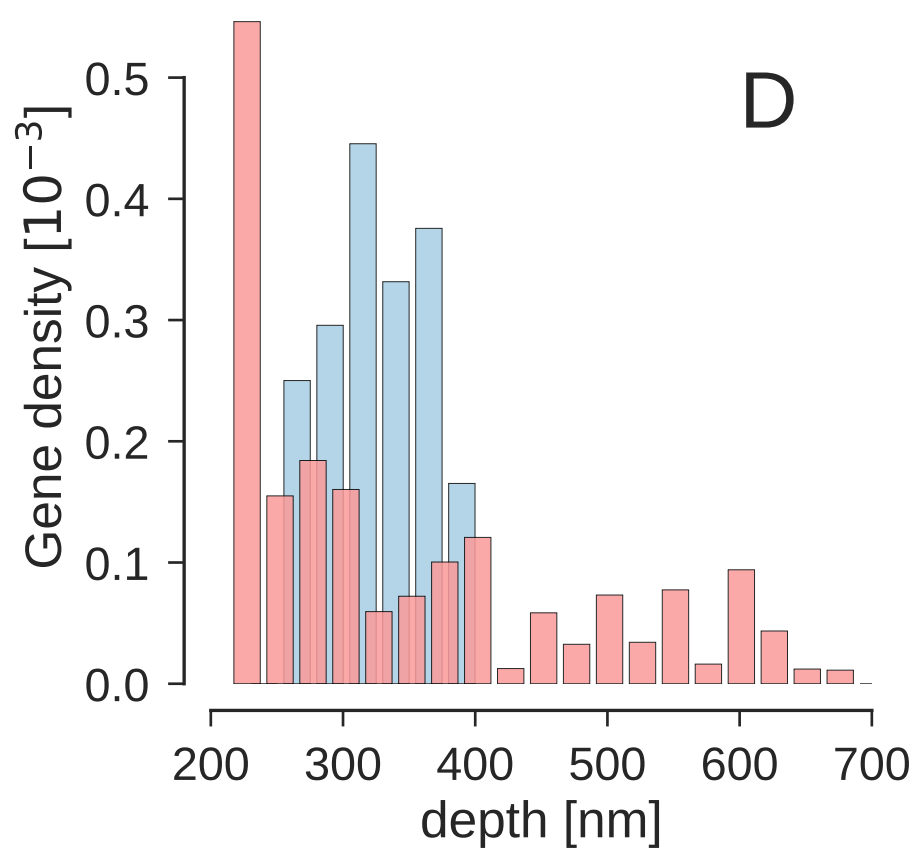

Supplement: S9 Fig — The cumulative number of trans-chromosomal contacts (A), lamin B1 (B) interaction scores, H3K4me3 (C) and gene density (D) are calculated as a function of depth for the prior ensemble based on chain connectivity, volume exclusion and the radius of gyration term (blue histogram) and the posterior ensemble incorporating the contact restraints (red histogram). Lamin B1 scores, H3K4me3 and gene density were obtained from the Supplementary Data in Nagano et al. [11]. (PDF) [file pcbi.1005292.s013.pdf]

X chromosome position [Mb]

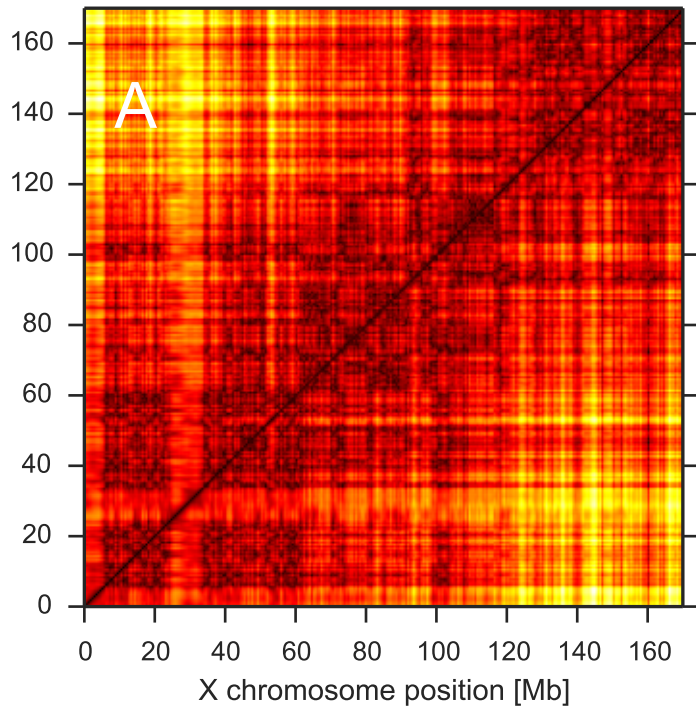

X chromosome position [Mb]

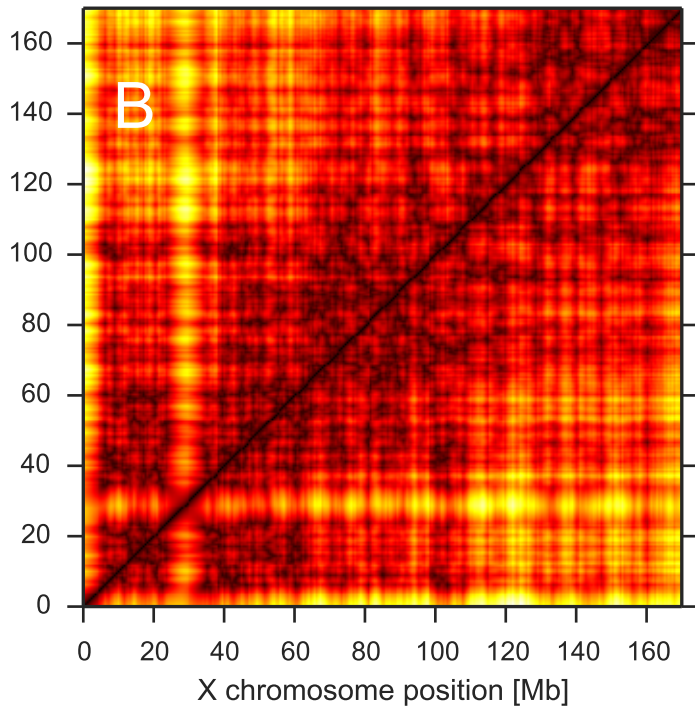

Supplement: S10 Fig — (PDF) [file pcbi.1005292.s014.pdf]

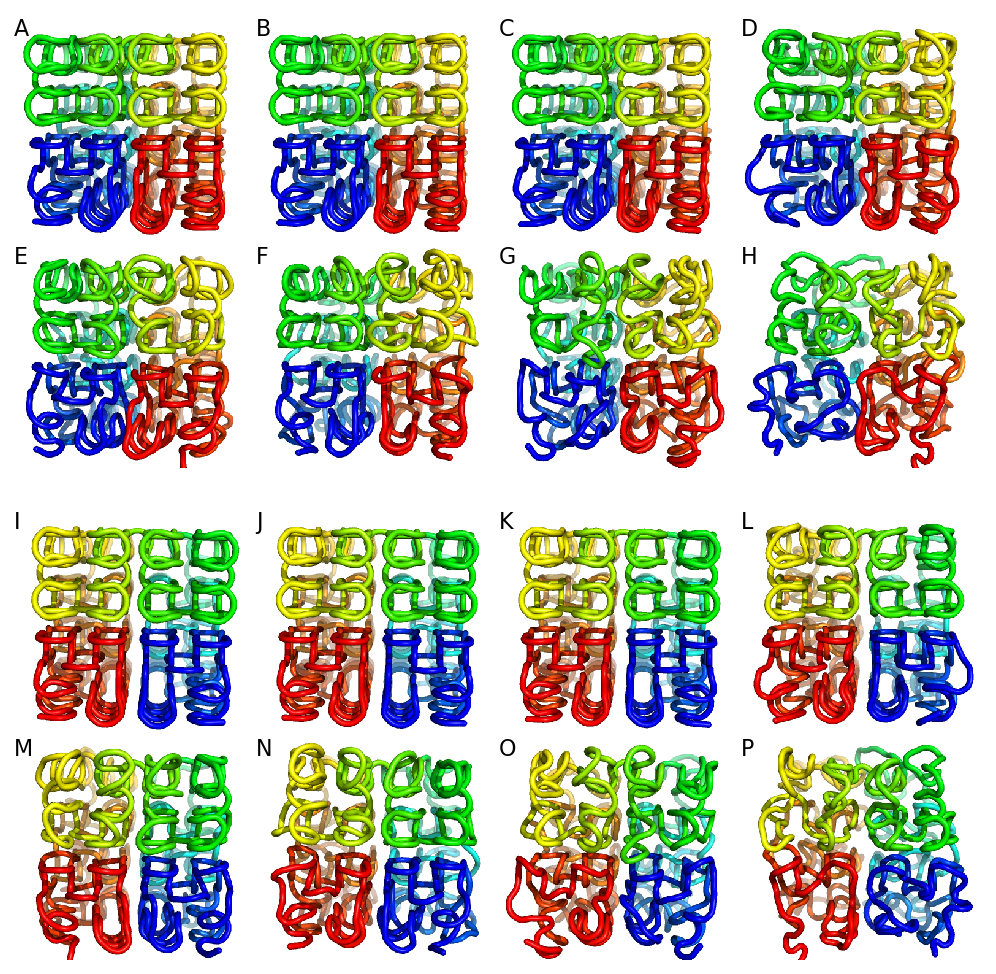

Supplement: S11 Fig — Top two rows show the original structure (A) and conformers inferred from contact data of different levels of sparsity (B-H). Corresponding mirror images are shown in the bottom rows (I-P). In all cases, both conformers are almost equally populated. Number of contacts used in reconstruction 3696 (B,J), 3326 (C,K), 1848 (D,L), 1478 (E,M), 1108 (F,N), 889 (G,O), 578 (H,P). (PNG) [file pcbi.1005292.s015.png]

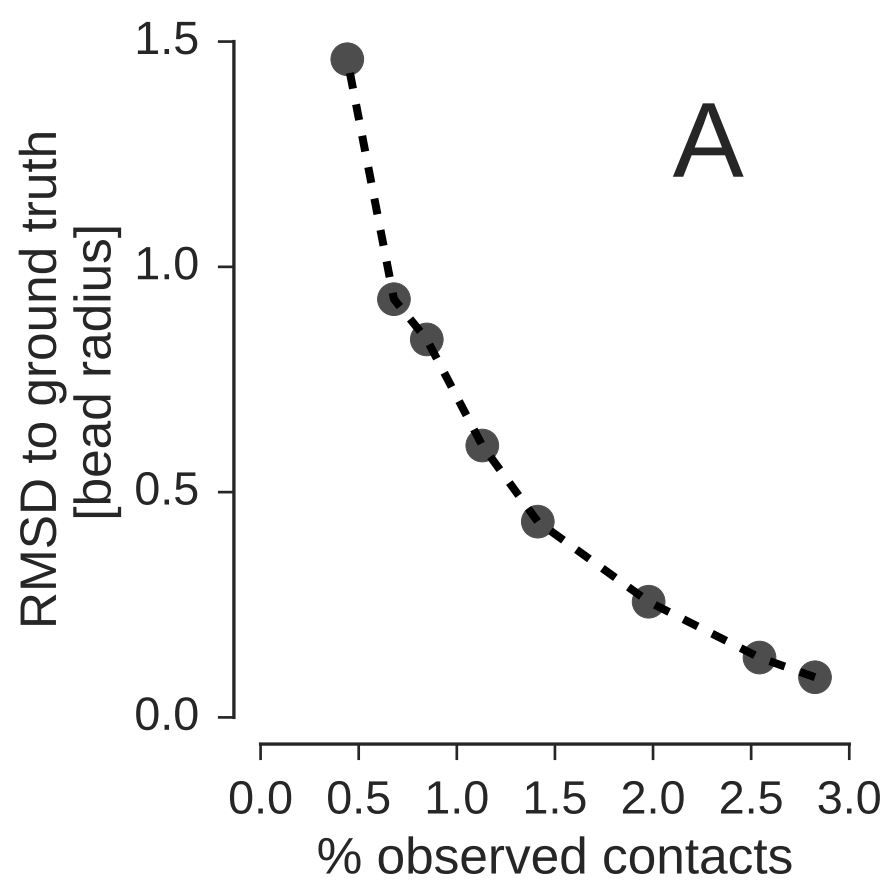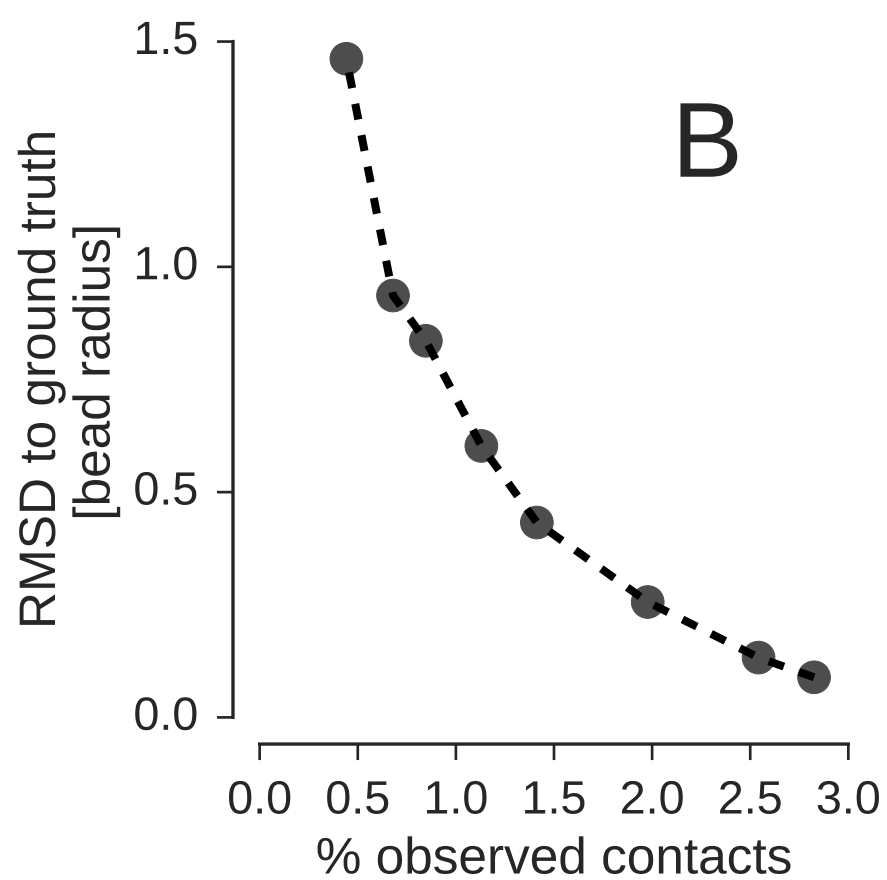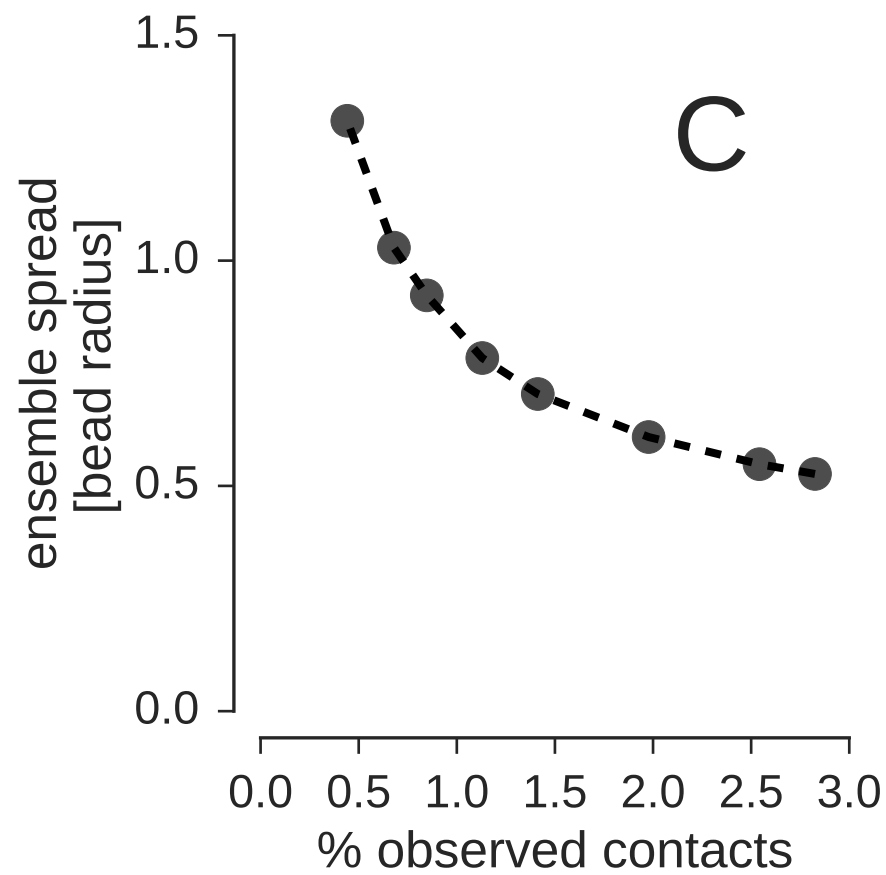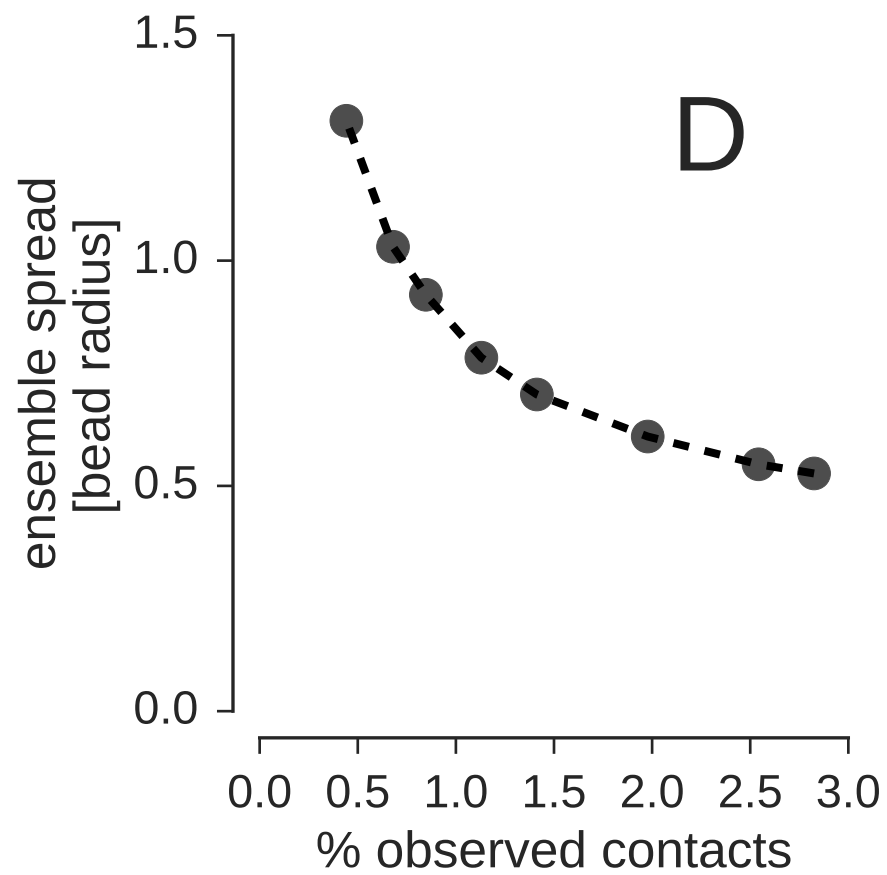

Supplement: S12 Fig — Tests were run on the 512-Hilbert curve with dwindling number of input data. Each ISD ensemble comprised two major clusters corresponding to the input curve and its mirror image. For each cluster, the accuracy was assessed by computing the RMSD to the input structure (A) / its mirror image (B). The precision or ensemble spread is defined as the average RMSD to the cluster center and shown in panels (C,D). (PDF) [file pcbi.1005292.s016.pdf]

A

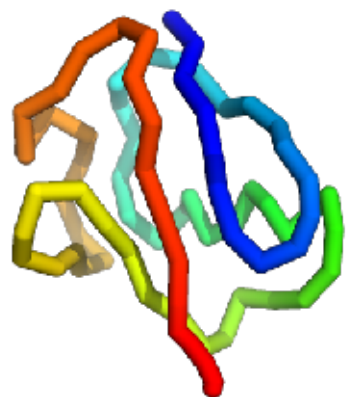

B

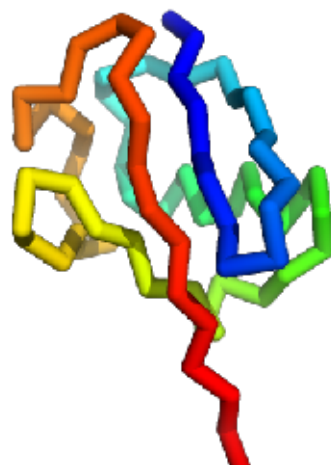

C

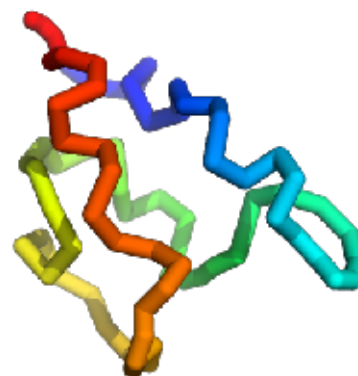

D

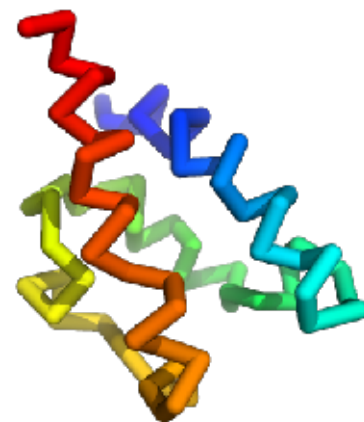

E

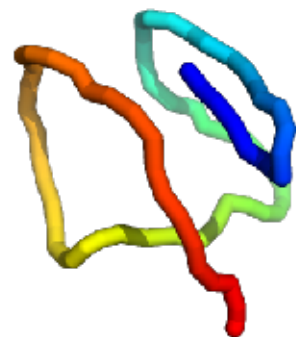

F

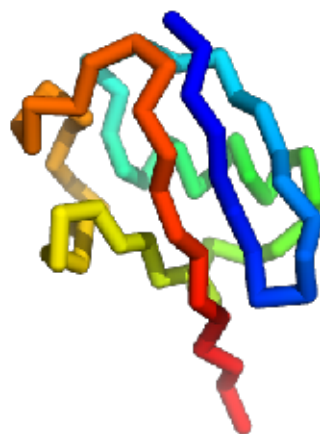

G

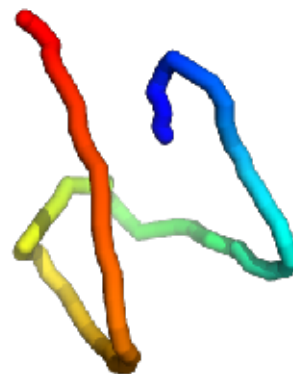

H

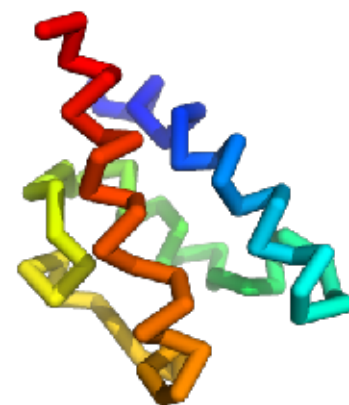

Supplement: S13 Fig — Ubiquitin is shown in the left panels, the HRDC domain is shown in the right panels. The top row shows the average bead models obtained from the mixed set of distances. The bottom row shows the average bead models obtained from mixed sets of sparse contacts. Ubiquitin models obtained with distance / sparse contact information are shown in panels (A) and (E). The ground truth is shown in panels (B) and (F). Models of the HRDC domain obtained with distance / sparse contact information are shown in panels (C) and (G). The ground truth is shown in panels (D) and (H). (PDF) [file pcbi.1005292.s017.pdf]

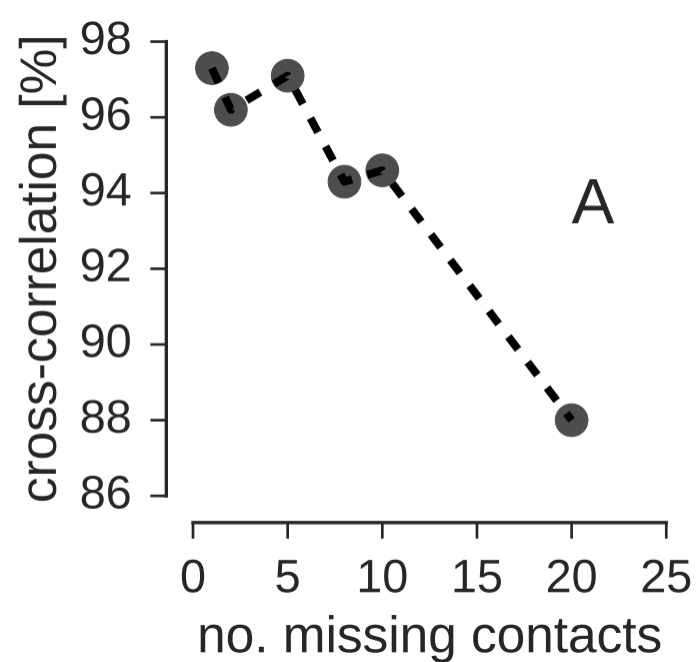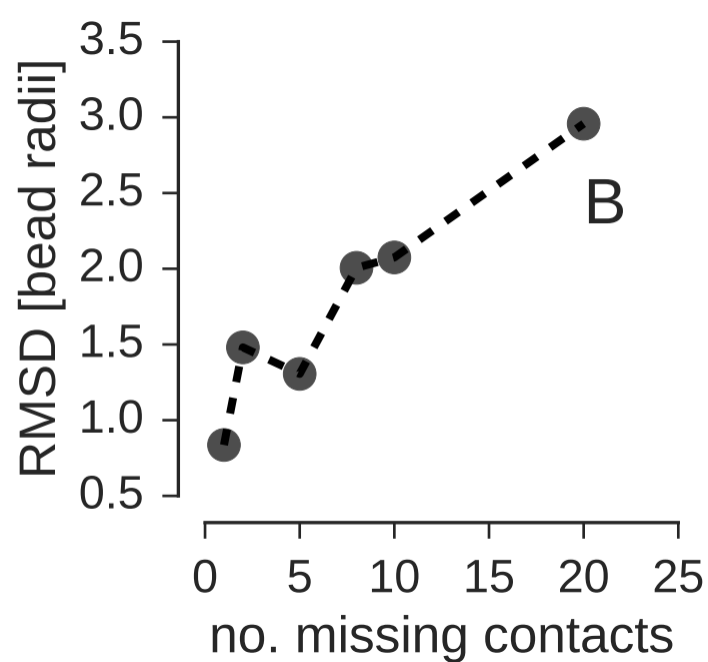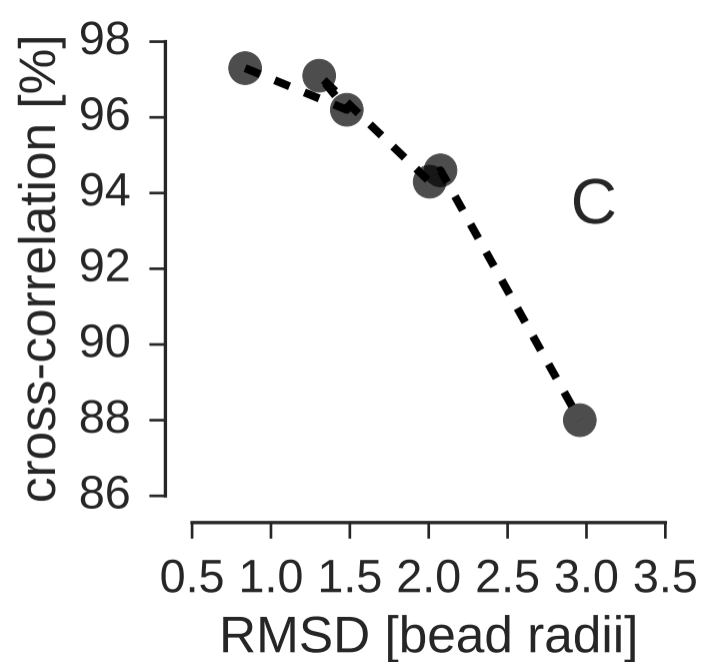

Supplement: S14 Fig — Structure ensembles were calculated with a reduced set of contacts where the n longest range contacts have been ignored. The ISD ensembles based on the reduced set of data were compared with the ensemble obtained with the full data set to study the robustness of the ISD approach and the significance of the distance correlations. (A) Cross-correlation between average distance matrices as a function of the number of missing long-range contacts. (B) RMSD between the average structures of the ensemble obtained with all contacts and reduced number of contacts. (C) Correlation between correlation of distance matrices and RMSD. (PDF) [file pcbi.1005292.s018.pdf]
