# Supplementary material for: Inferential Structure Determination of Chromosomes from Single-Cell Hi-C Data
Source: PLoS Comput Biol. 2016 Dec 27;12(12):e1005292. doi: 10.1371/journal.pcbi.1005292 (PMC5226817; doi:10.1371/journal.pcbi.1005292)
Supplement: S1 Table — (PDF) [file pcbi.1005292.s002.pdf]

Estimated model evidence

Prior probability	model evidence	model evidence per data point
Quartic repulsion	-2489	-5.68
Quartic repulsion + FISH	-2363	-5.39
Lennard-Jones	-1950	-4.45
Lennard-Jones + FISH	-1859	-4.24

Table S1: Model evidence based on contact likelihood.
