# Supplementary material for: Inferential Structure Determination of Chromosomes from Single-Cell Hi-C Data
Source: PLoS Comput Biol. 2016 Dec 27;12(12):e1005292. doi: 10.1371/journal.pcbi.1005292 (PMC5226817; doi:10.1371/journal.pcbi.1005292)
Supplement: S1 Appendix — (PDF) [file pcbi.1005292.s005.pdf]

Supplementary Appendix

Full inference of distance-based models

Bayesian inference also allows us to estimate both parameters, γ and σ, simultaneously. The ensemble obtained with free parameters appears to be the blurred version of the ensemble with fixed data weight (see Fig. A1 for a comparison of the average distance matrices). The parameters of the lognormal model are shown in Figure A2. Both γ and the weight $w = 1/\sigma^2$ are estimated to be smaller compared to the values obtained with fixed w. Figure A3 shows a comparison of the energies, log likelihoods, radii of gyration and other quantities for the simulation with fixed and variable σ.

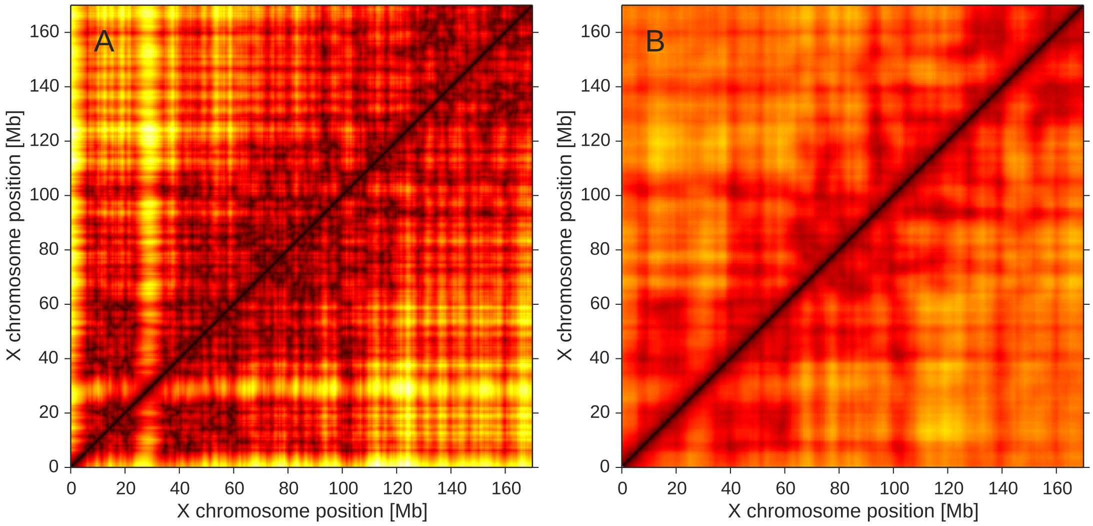

Fig A1: Comparison of distance-based structure ensembles assuming a lognormal error model. **(A)** Average distance matrix with fixed data weight $w = \sigma^{-2} = 500$. **(B)** Average distance matrix with free $w = \sigma^{-2}$ and distance scale γ.

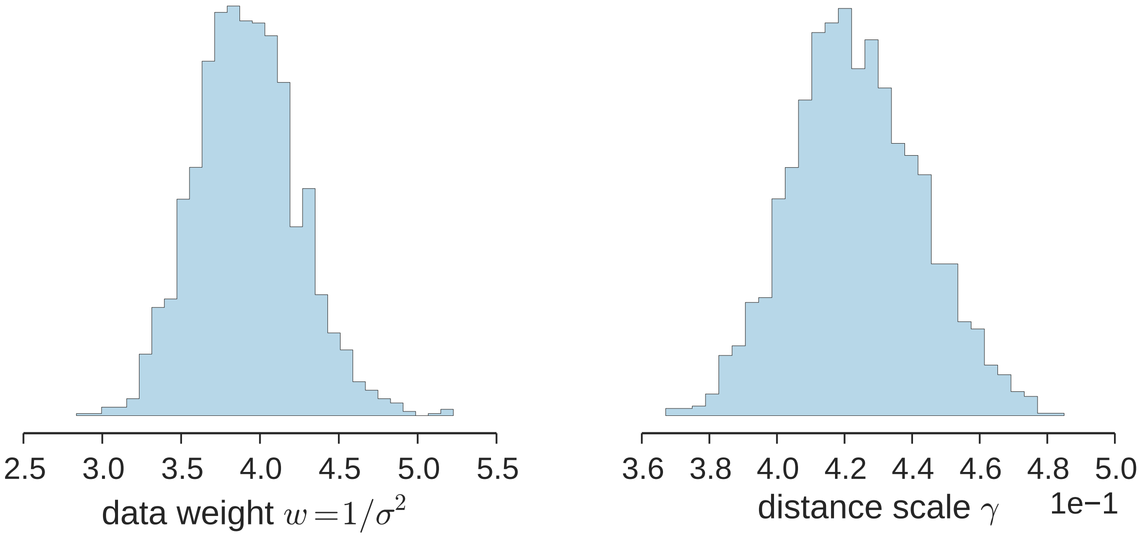

Fig A2: Estimated model parameters for the lognormal model.

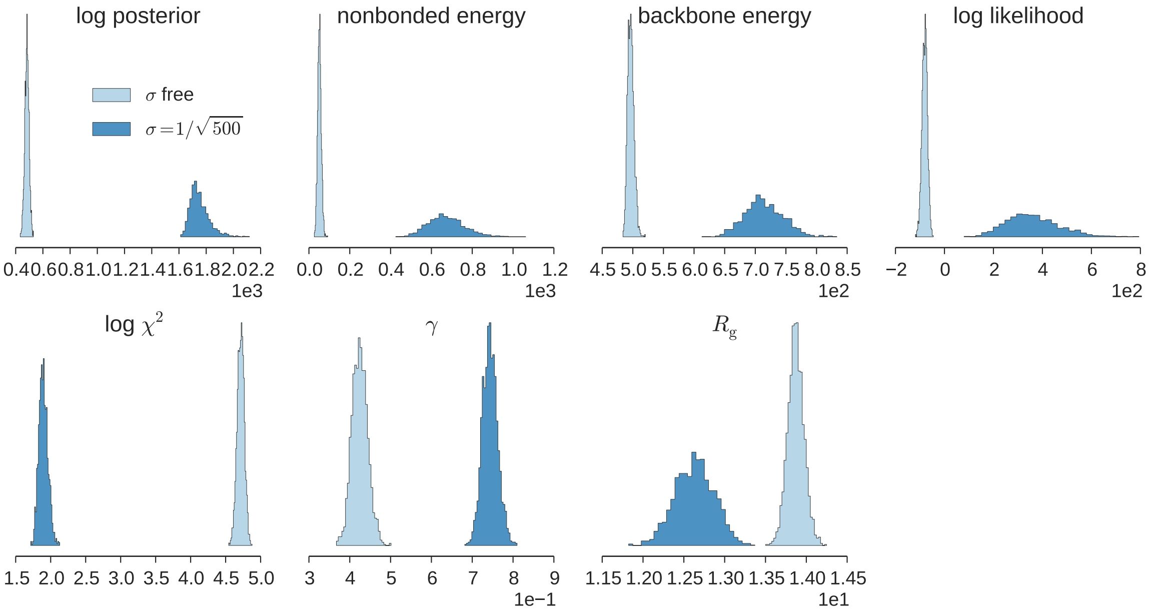

Fig A3: Comparison of ensembles calculated with fixed and variable data weight σ^{-2}.
