# Supplementary material for: Inferential Structure Determination of Chromosomes from Single-Cell Hi-C Data
Source: PLoS Comput Biol. 2016 Dec 27;12(12):e1005292. doi: 10.1371/journal.pcbi.1005292 (PMC5226817; doi:10.1371/journal.pcbi.1005292)
Supplement: S2 Table — Shown are the correlation coefficients between the average distance matrices (in %) for structure ensembles obtained from cell 1 to cell 6. Lower diagonal: ISD ensemble vs ISD ensemble. Upper diagonal: ISD ensemble vs ensemble by Nagano et al. Diagonal (shown in bold face): ISD ensemble vs ensemble by Nagano et al. (PDF) [file pcbi.1005292.s008.pdf]

Correlation between average distance matrices

	cell 1	cell 2	cell 3	cell 4	cell 5	cell 6
cell 1	**85.2**	49.5	57.5	58.2	47.6	49.9
cell 2	46.2	**92.1**	52.2	51.0	40.9	37.5
cell 3	54.0	46.7	**91.5**	49.5	51.0	43.0
cell 4	55.1	45.5	45.4	**91.1**	46.8	45.1
cell 5	44.5	36.0	46.2	42.5	**70.6**	33.5
cell 6	50.2	33.2	42.5	44.1	36.3	**91.3**

Table S2: Comparison of distance matrices. Shown are the correlation coefficients between the average distance matrices (in %) for structure ensembles obtained from cell 1 to cell 6. Lower diagonal: ISD ensemble vs ISD ensemble. Upper diagonal: ISD ensemble vs ensemble by Nagano *et al.* Diagonal (shown in bold face): ISD ensemble vs ensemble by Nagano *et al.*
