# Supplementary material for: Inferential Structure Determination of Chromosomes from Single-Cell Hi-C Data
Source: PLoS Comput Biol. 2016 Dec 27;12(12):e1005292. doi: 10.1371/journal.pcbi.1005292 (PMC5226817; doi:10.1371/journal.pcbi.1005292)
Supplement: S3 Table — Percentage of restraints from different cells (columns) that are violated in structure ensembles (rows) with a tolerance of a/8. (PDF) [file pcbi.1005292.s009.pdf]

Violations of restraints from six different cells

	restraints from cell 1	cell 2	cell 3	cell 4	cell 5	cell 6
ensemble based on cell 1	**0.0**	64.5	73.2	71.1	69.8	69.0
cell 2	73.2	**0.0**	74.5	79.6	70.8	71.0
cell 3	75.5	68.9	**0.0**	79.0	70.3	69.1
cell 4	70.6	65.2	73.7	**0.0**	68.8	69.6
cell 5	73.4	68.8	72.5	79.3	**0.0**	71.7
cell 6	76.1	68.3	77.6	79.1	72.1	**0.0**

Table S3: Percentage of restraints from different cells (columns) that are violated in structure ensembles (rows) with a tolerance of $a/8$.
