# Supplementary material for: Inferential Structure Determination of Chromosomes from Single-Cell Hi-C Data
Source: PLoS Comput Biol. 2016 Dec 27;12(12):e1005292. doi: 10.1371/journal.pcbi.1005292 (PMC5226817; doi:10.1371/journal.pcbi.1005292)
Supplement: S4 Table — Tests for the calculation of a single structure were done on a standard laptop with 2.90GHz Core i7 8-core processor with 16 GB memory. At a resolution of 500 kb, the test consisted of 1000 steps of HMC starting from an extended structure. At a resolution 50 kb, 10000 steps of HMC were run starting from an extended structure. To compute the posterior ensemble of the X chromosome, we ran the replica-exchange algorithm on a computer cluster using 50 CPUs. At a resolution of 500 kb, we simulated 200 replica transitions where each transition consisted of 10 steps of HMC each using 250 leapfrog integration steps. At a resolution of 50 kb, we ran 1000 replica transitions on 50 CPUs. It is possible to shortcut the computation of the high-resolution structure by starting from a low resolution model. The initial structure of the high-resolution model is obtained by using a 3D spline interpolation with 10-fold higher sampling. For the single structure calculation, it is sufficient to reduce the number of HMC steps to 1000. In case of the replica simulation, the correct ensemble is obtained after 200 replica transitions. The corresponding computation times are indicated by “500 kb + 50 kb” in the S4 Table. (PDF) [file pcbi.1005292.s019.pdf]

Computation times

ISD result	Resolution	Computation time
single structure	500 kb	52.1 s
single structure	50 kb	6302.4 s
single structure	500 kb + 50 kb	352.2 s
ensemble	500 kb	1.8 h
ensemble	50 kb	56 h
ensemble	500kb + 50 kb	5 h

Table S4: Tests for the calculation of a single structure were done on a standard laptop with 2.90GHz Core i7 8-core processor with 16 GB memory. At a resolution of 500 kb, the test consisted of 1000 steps of HMC starting from an extended structure. At a resolution 50 kb, 10000 steps of HMC were run starting from an extended structure. To compute the posterior ensemble of the X chromosome, we ran the replica-exchange algorithm on a computer cluster using 50 CPUs. At a resolution of 500 kb, we simulated 200 replica transitions where each transition consisted of 10 steps of HMC each using 250 leapfrog integration steps. At a resolution of 50 kb, we ran 1000 replica transitions on 50 CPUs. It is possible to shortcut the computation of the high-resolution structure by starting from a low resolution model. The initial structure of the high-resolution model is obtained by using a 3D spline interpolation with 10-fold higher sampling. For the single structure calculation, it is sufficient to reduce the number of HMC steps to 1000. In case of the replica simulation, the correct ensemble is obtained after 200 replica transitions. The corresponding computation times are indicated by "500 kb + 50 kb" in the S4 Table.
